# Dynamic regulation of secondary metabolites and agarwood aroma compounds in *Aquilaria sinensis* by the endophytic fungus NSZJ-CX-22 revealed through metabolomics and GC–MS

**DOI:** 10.3389/fpls.2026.1750077

**Published:** 2026-04-10

**Authors:** Tianjiao Ma, Hui Meng, Jianhe Wei, Yun Yang

**Affiliations:** 1 Institute of Medicinal Plant Development, Chinese Academy of Medical Sciences & Peking Union Medical College, Beijing, China; 2 Hainan Provincial Key Laboratory of Resources Conservation and Development of Southern Medicine, Hainan Branch of the Institute of Medicinal Plant Development, Chinese Academy of Medical Sciences and Peking Union Medical College, Haikou, China

**Keywords:** agarwood, *Aquilaria sinensis*, *Arthrinium* sp., endophytic fungus, GC–MS, secondary metabolites, temporal dynamics, widely targeted metabolomics

## Abstract

**Background:**

Agarwood, a valuable aromatic resin from *Aquilaria* species, is prized for its distinctive fragrance and medicinal properties. However, natural agarwood formation is slow, and the quality of artificially induced agarwood remains unstable. While endophytic fungi can stimulate resin accumulation by activating metabolic pathways, the temporal dynamics of metabolite synthesis, the mechanisms of pathway convergence, and the quantitative relationships between precursor consumption and aromatic compound production remain poorly understood, limiting the development of standardized bioinduction systems.

**Methods:**

We employed a sterile *A. sinensis* sawdust medium and inoculated the high-efficiency agarwood-inducing endophytic fungus NSZJ-CX-22 to construct a controllable model system. We systematically evaluated the dynamic variation patterns of metabolite profiles at four culture time points (7th, 14th, 21st, and 28th days post-inoculation) using widely targeted metabolomics (LC-MS/MS) and gas chromatography–mass spectrometry (GC–MS). We applied multivariate statistical approaches, including principal component analysis (PCA) and orthogonal partial least squares discriminant analysis (OPLS-DA), to decipher the metabolic dynamics. Additionally, we performed KEGG enrichment analysis to identify key metabolic pathways.

**Results:**

We identified 14,784 metabolites, including 118 agarwood-characteristic sesquiterpene skeletons and 2-(2-phenylethyl)chromones (PECs). Three core biosynthetic pathways—flavonoid, phenylpropanoid, and terpenoid—converge through shared precursors (acetyl-CoA and malonyl-CoA) and branch-point enzymes (CHS, TPS, PKS), orchestrated by jasmonic acid (JA)-like signals. The metabolic trajectory exhibited a triphasic pattern: nutrient mobilization (0–7d), biosynthetic burst with peak sesquiterpene/chromone accumulation at day 14, and defensive restructuring (21–28d) with P450-mediated modifications. Quantitative correlation analysis revealed significant negative relationships (|r| ≈ 0.63–0.74, FDR<0.05) between precursor depletion and agarwood compound synthesis. Volatile profiling identified 116 compounds, with aromatic compounds (42) and sesquiterpenes (5) as key odorants, accumulating in stage-specific patterns aligned with precursor flux redirection.

**Conclusion:**

This study establishes a “precursor pool–pathway convergence–temporal programming” framework for fungal agarwood biogenesis. The integrated multi-omics approach quantitatively links substrate consumption to product synthesis. It provides actionable guidance for optimized bioproduction: segmented harvesting (sesquiterpenes at 7–14d, chromones avoiding the day-21 energy trough), JA-signal priming in the early phase, and P450-enhancement strategies in the late phase for structural diversification. These findings advance mechanistic understanding and enable scalable, quality-controlled agarwood biomanufacturing.

## 1 Introduction

Agarwood is a highly valued aromatic dark resin produced by *Aquilaria* and *Gyrinops* species, renowned for its distinctive fragrance and multiple biological activities. Historically used in perfumes, essential oils, traditional medicine, and religious rituals (Persoon, 2007; Zhang et al., 2012; Liu et al., 2013, Liu et al, 2017; Rishu Kalra and Nutan Kaushik, 2017; Al-Hindi et al., 2018; Wang et al., 2018, Wang et al., 2019; Tan et al., 2019), agarwood exhibits pharmacological properties including antibacterial, anti-inflammatory, sedative, anxiolytic, and digestive-promoting effects (Li et al., 2021, Li et al., 2024; Zhang et al., 2024; Pharmacopoeia, 2020). Chemically, its signature aroma derives from sesquiterpenes and 2-(2-phenylethyl)chromones (PECs), along with volatile aromatic compounds (Fazila and Halim, 2012; Ismail et al., 2017; Liu et al., 2013; Li et al., 2021; Ma et al., 2021; Zhang et al., 2025b). However, natural agarwood formation is slow and rare. At the same time, escalating commercial demand has driven overexploitation of wild populations, prompting the inclusion of *Aquilaria* and Gyrinops on the Convention on International Trade in Endangered Species of Wild Fauna and Flora (CITES) list to protect these endangered resources (Barden et al., 2000; Compton and Ishihara, 2004; López-Sampson and Page, 2018; Rasool and Mohamed, 2016; Raymakers, 2006; Turjaman et al., 2016).

Agarwood formation represents an injury-induced pathological defence response in *Aquilaria sinensis*. Although artificial induction methods—including physical wounding, chemical treatment, and biological inoculation—have been developed, the quality and yield of induced agarwood remain unstable (Wu and Yu, 2023). Among these approaches, fungal bioinduction has attracted considerable interest because numerous endophytic fungi can trigger resin accumulation and produce bioactive metabolites (Azren et al., 2019; Faizal et al., 2020; Han et al., 2014; Laurence, 2013; Monggoot et al., 2017; Qi et al., 1998; Subasinghe et al., 2019; Tian et al., 2013; Tibpromma et al., 2021; Zhang et al., 2025a). Species such as *Colletotrichum gloeosporioides* (Tian et al., 2013), *Botrysphaeria* sp (Tian et al., 2013), *Fusarium solani* (Faizal et al., 2017; Chen et al., 2017), *Lasiodiplodia theobromae* (Chen et al., 2017), *Nodulisporium* sp (Cui et al., 2013), and *Penicillium variabile* (Wu et al., 2010) variabile stimulate the biosynthesis of sesquiterpenes, PECs, chromones, and other characteristic metabolites, providing a biochemical explanation for the richness and complexity of the aroma in fungus-induced agarwood.

Mechanistically, fungal inoculation triggers rapid activation of the host defence network. Wounding and fungal infection elevate enzyme activities, increase phytohormone levels, including jasmonic acid (JA), salicylic acid (SA), and 1-aminocyclopropane-1-carboxylic acid (ACC), promote the accumulation of osmoprotectants, and reshape microbial community structure (Fang et al., 2024; Li et al., 2022; Liu et al., 2021, Liu et al., 2024). These coordinated responses drive the biosynthesis and accumulation of phenolics, chromones, and terpenoids (Mohamed et al., 2010). During fungal colonization, starch is progressively degraded into non-starch polysaccharides and phenolic precursors, feeding pathways that generate sesquiterpenes and PECs (Cui et al., 2013). Early defence activation enhances fatty acid oxidation, markedly increasing JA, SA, and ACC levels, which in turn upregulate key biosynthetic genes such as sesquiterpene synthases (TPS) and polyketide synthases (PKS) (Küpper et al., 2009; Lv et al., 2019; Xu et al., 2017; Zhang et al., 2022a, Zhang et al., 2022b). Furthermore, endophytic fungi secrete stress-related metabolites and signalling molecules that modulate host defence gene expression (Chaudhary et al., 2022; Fadiji et al., 2023; Schulz et al., 2002; Spaepen and Vanderleyden, 2011), further reinforcing resin formation (Azren et al., 2019; Liu et al., 2024; Ma et al., 2021).

Plant sesquiterpenes biosynthesis proceeds mainly through the 2-C-methyl-D-erythritol 4-phosphate pathway (MEP) and mevalonate pathway (MVA), generating farnesyl pyrophosphate (FPP) as the precursor, while cytochrome P450 monooxygenases (CYP450s) and TPS enzymes create extensive structural diversity (Pulido et al., 2012). Fungal metabolites, such as frabenol and squalene, have also been implicated in the formation of aromatic compounds and sesquiterpenes (Bhuiyan et al., 2009; Okudera and Ito, 2009; Pulido et al., 2012; Sen et al., 2017). PECs, occurring in only a few plant species, are tightly linked to defence-associated pathways; *Fusarium solani*, for example, can enhance chromone accumulation by regulating chalcone synthase (CHS) (Chen et al., 2017; Shivanand et al., 2022). These findings highlight the central role of fungal–host signalling in shaping the secondary metabolic landscape of agarwood formation.

Importantly, three key secondary metabolic pathways—flavonoid, phenylpropanoid, and isoflavonoid biosynthesis—converge at multiple biochemical nodes to support agarwood compound synthesis: (1) Shared precursors: acetyl-CoA and malonyl-CoA serve as core substrates for both sesquiterpenes (via MVA/MEP) and PECs (via PKS); (2) Branch-point enzymes: CHS acts as a key junction connecting phenylpropanoid and flavonoid pathways, directly modulating conversion of phenylalanine-derived precursors to chromone skeletons; TPS diverts isoprenoid precursors from general terpenoid metabolism to agarwood-specific sesquiterpene synthesis; (3) Temporal orchestration: These convergent networks form the molecular basis for coordinated agarwood aroma compound synthesis.

Despite significant progress in understanding the positive regulatory effects of endophytic fungi on agarwood formation, artificially induced agarwood remains of low quality and is unstable. This predicament primarily stems from an insufficient understanding of the time-dependent dynamics of metabolite accumulation during the fungus-host interaction. This knowledge gap regarding the convergence relationships among core secondary metabolic pathways and flux allocation patterns of precursor metabolites. While existing studies have identified key agarwood-related pathways, they have not clearly defined: (i) directional allocation mechanisms of shared precursors between pathways; (ii) temporal regulation characteristics of branch-point enzymes; (iii) quantitative correlations between precursor metabolite depletion and aromatic compound synthesis. These knowledge gaps limit the development of efficient, standardized fungal-induced agarwood production systems.

Based on extensive field screening, this study selected the endophytic fungus strain NSZJ-CX-22 with strong induction potential. A sterile *A. sinensis* sawdust medium inoculation model (inoculated with NSZJ-CX-22) was established, followed by the application of widely targeted metabolomics (LC-MS/MS) combined with gas chromatography–mass spectrometry (GC–MS) technology. A time-course dynamic analysis of secondary metabolite profiles was conducted at four culture time points (7th, 14th, 21st, and 28th day post-inoculation). Our objectives are to: (i) reveal convergence mechanisms and key regulatory nodes in core secondary metabolic pathways; (ii) identify the most significantly depleted precursor metabolite categories and their quantitative correlations with aromatic compound synthesis; (iii) elucidate temporal accumulation patterns of fungal metabolites and their unique metabolic regulatory mechanisms. This study provides a precise theoretical and experimental foundation for efficient, standardized production of fungus-induced agarwood.

## 2 Materials and methods

### 2.1 General experimental procedures

#### 2.1.1 Fungal samples

**Figure 1:**
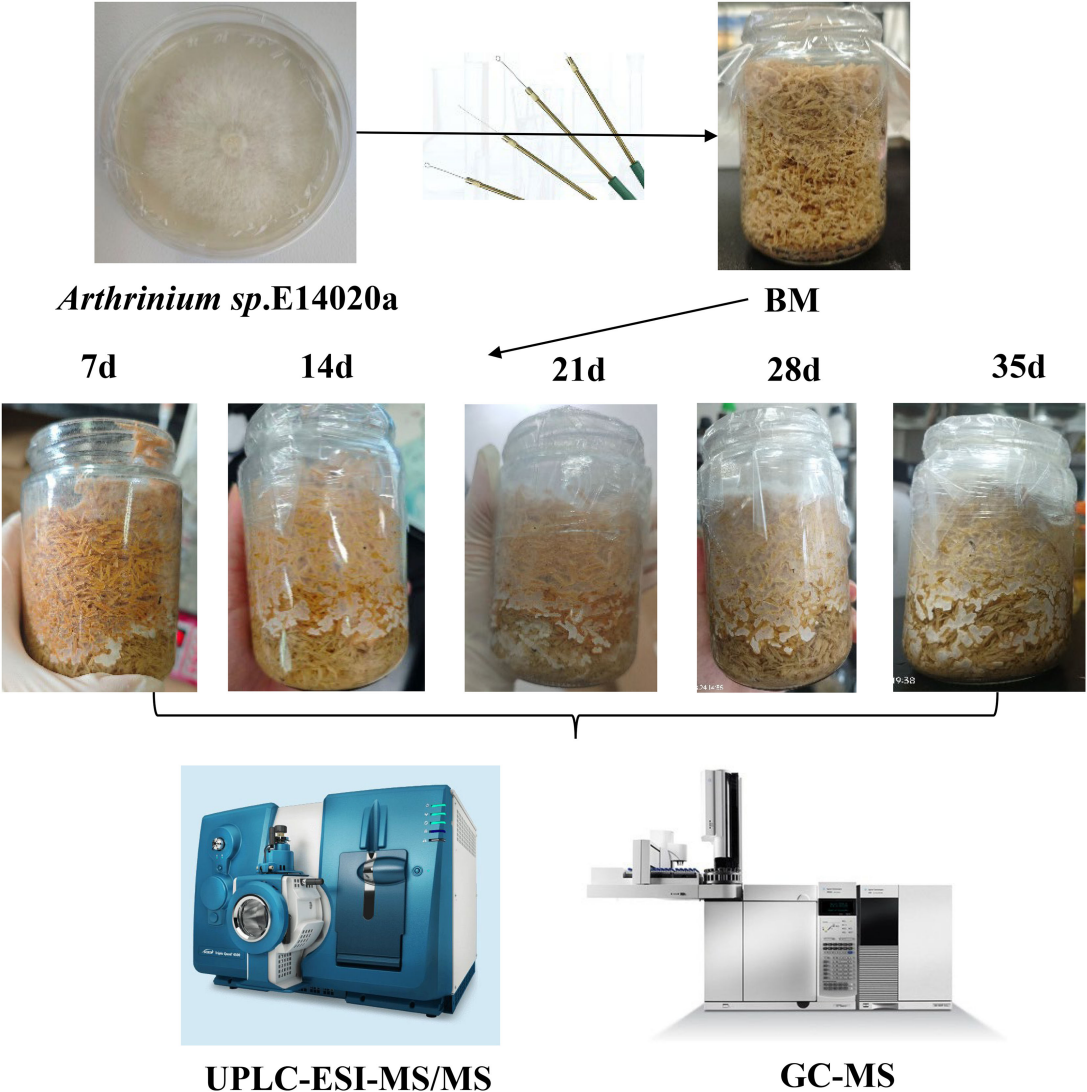
Schematic workflow for fungal cultivation and downstream metabolic analysis. An endophytic fungus was inoculated into a prepared white agarwood wood−chip substrate and cultivated at five time points. Since metabolite accumulation on the surface of the medium at 28d and 35d appeared almost identical, a fermentation duration of 28 days was chosen. After fermentation, biomass was harvested, and the extracts were analysed via non−targeted metabolomics and GC−MS to characterise the fermentation products.

The agarwood endophytic fungus (*Arthrinium* sp. E14020a) NSZJ-CX-22 (**Figure 1**) was deposited in the China General Microbiological Culture Collection Centre (CGMCC No. 42489) and the Laboratory of Hainan Branch Institute of Medicinal Plant Development, Chinese Academy of Medical Sciences (No. AS22). Phylogenetic identification based on internal transcribed spacer (ITS) sequence analysis (GenBank Accession No.: PX671043) confirmed the strain as *Arthrinium* sp. E14020a. Results of microscopic identification (ECLIPSE 80i Biological Microscope, Nikon Corporation, Japan) demonstrated that the strains exhibited a slender and branched filamentous structure, with septa dividing the mycelia into multiple cells. The arthrospores were cylindrical, with distinct connections between adjacent spores (**Supplementary Figure E**). Accordingly, the sterilized *Aquilaria sinensis* sawdust medium without inoculation was used as the blank control.

This strain was isolated from the xylem-resinous portion of a three-year-old *A. sinensis* (Lour.) Gilg. The plant was treated with the whole-wood agarwood-inducing technology in Hainan Province, using the traditional tissue isolation method. Specifically, surface sterilisation was performed with 75% ethanol and 10% sodium hypochlorite as disinfectants. After rinsing off residual disinfectants with sterile water, the excess moisture was blotted dry with sterile filter paper. Subsequently, the resinous and non-agarwood-formed portions were cut into small pieces with a sterile scalpel and inoculated onto potato dextrose agar (PDA) medium supplemented with 50 mg/L ampicillin. The inoculated plates were sealed and incubated at 28 °C for 3–5 days. After mycelial emergence, purification was performed using the hyphal tip picking method until pure single colonies were obtained. The purified strains were preserved in glycerol and stored in an ultra-low temperature freezer at −80 °C for subsequent use.

#### 2.1.2 Culture media, culture methods, and other reagents

The cryopreserved strains were thawed at room temperature and cultured on PDA medium at 28 °C for 5 days. Subsequently, 5-mm-diameter mycelial plugs were excised using a sterile puncher and inoculated into the sterilised, cooled *A. sinensis* sawdust medium (g/L: dried *A. sinensis* sawdust 480.0, sterile water 720.0). The cultures were incubated in a dark environment at 28 °C for 7, 14, 21, and 28 days, respectively. The morphological characteristics of the cultures at the corresponding time points are shown in **Figure 1**, with three biological replicates per time point. Since no significant difference in metabolite accumulation on the medium surface was observed between the 35th and the 28th days of incubation, the culture period was ultimately set at 28 days.

All reagents used in this study met the standards of analytical grade or chromatographic grade. PDA medium was purchased from Qingdao Hope Bio-Technology Co., Ltd. Anhydrous ethanol, ethyl acetate, and n-hexane were of analytical grade. Chromatographic-grade ethyl acetate was supplied by Shanghai ANPEL Laboratory Technologies Inc. Methanol, acetonitrile, and isopropanol were procured from CNW Technologies, while acetic acid was purchased from Sigma-Aldrich Corporation.

### 2.2 Widely targeted metabolomics analysis

#### 2.2.1 Sample extraction and preparation

Each fermentation product and the corresponding blank medium were first soaked in 200 mL of anhydrous ethanol for one hour, followed by ultrasonic extraction for 30 min. This extraction procedure was repeated three times, and the extracts were combined to a final volume of 600 mL. The combined extracts were then concentrated under reduced pressure and stored at −80 °C until further analysis. Before analysis, samples were thawed on ice and vortexed for 30 s to ensure homogeneity. An aliquot of 200 μL was transferred to a PE tube, evaporated to dryness, and reconstituted in 200 μL of 1× extraction solution (methanol: water = 4:1, containing the internal standard). The mixture was vortexed for 30 seconds and then sonicated in an ice-water bath for 10 minutes. Samples were then centrifuged at 12,000 rpm and 4 °C for 15 minutes. The resulting supernatant was filtered through a 0.22 μm membrane and analysed by UPLC–MS/MS analysis. For quality control (QC), equal volumes from each sample were combined to prepare a QC sample.

#### 2.2.2 UPLC-MS/MS conditions

The sample extracts were analysed using a UPLC–ESI–MS/MS system (UPLC: Thermo Fisher Scientific Vanquish; MS: Thermo Fisher Scientific Stellar). UPLC conditions: Chromatographic separation was performed on a UPLC Kinetex C18 column (2.1 mm × 50 mm, 2.6 μm). The mobile phase consisted of solvent A (0.01% aqueous acetic acid) and solvent B (50% acetonitrile/isopropanol). The gradient elution program (total 6 min) was as follows: 99% A and 1% B for 0.5 min; a linear gradient to 1% A and 99% B over 4 min, held for 0.5 min; then returned to 99% A and 1% B within 0.05 min and maintained for 0.95 min. The flow rate was set at 0.3 mL/min, the column temperature at 25 °C, and the injection volume was two μL. The column effluent was directed to an ESI-triple quadrupole linear ion trap mass spectrometer (Stellar), and targeted metabolite analysis was performed using Parallel Reaction Monitoring (PRM) mode.

### 2.3 GC-MS analysis

#### 2.3.1 Sample extraction and preparation

Each solid fermentation product and the corresponding blank medium were first soaked in 300 mL of ethyl acetate for one hour, followed by ultrasonic extraction three times, each for 30 min. The three extracts were combined and brought to a final volume of 900 mL with ethyl acetate. An aliquot of 45 mL of the extract was then subjected to n-hexane partitioning, and the ethyl acetate phase was collected and evaporated to dryness. The remaining sample was dissolved in 6 mL of chromatographic-grade ethyl acetate and filtered through a 0.22 μm membrane for subsequent GC–MS analysis. For quality control (QC), equal volumes from each sample were combined to prepare a QC sample.

#### 2.3.2 GC-MS conditions

Chromatographic analysis was performed using an HP-5MS capillary column (5% phenyl methylpolysiloxane, 30 m × 250 μm × 0.25 μm). The injector temperature was set at 240 °C. High-purity helium was used as the carrier gas at a flow rate of 1 mL·min^-^¹. Samples (1 μL) were injected in splitless mode. The oven temperature program was as follows: initially 60 °C for 2 min; ramped at 10 °C·min^-^¹ to 160 °C and held for 1 min; then increased at 2 °C·min^-^¹ to 230 °C and held for 1 min; finally ramped at 4 °C·min^-^¹ to 300 °C and held for 2 min, with a total run time of 68.5 min. Mass spectrometry conditions were set as follows: electron ionisation (EI) source at 230 °C, ionisation energy 70 eV; quadrupole temperature 150 °C; MS interface temperature 300 °C. Data were acquired in full-scan mode over the m/z range 50–500.

### 2.4 Multivariate statistical analysis and network visualisation

Raw data were first preprocessed and subjected to quality control to ensure high-quality and reproducible results. Subsequently, statistical analyses were performed, including both multivariate and univariate approaches. Multivariate analysis, comprising unsupervised principal component analysis (PCA) and hierarchical clustering, was employed to evaluate the metabolic variation patterns of the endophytic fungus across different cultivation time points. For pairwise comparisons, differential metabolites were identified based on variable importance in projection (VIP) values ≥ 1.0, from the orthogonal partial least squares discriminant analysis (OPLS-DA) model, and absolute Log2 fold change (|Log_2_FC|) values ≥ 1.0, allowing for the detection of significantly altered metabolic features.

### 2.5 Metabolites annotation and KEGG pathway enrichment analysis

Identified metabolites were first annotated using the Kyoto Encyclopedia of Genes and Genomes (KEGG) Compound (http://www.kegg.jp/kegg/compound/) and KEGG Pathway (http://www.kegg.jp/kegg/pathway.html) (Kanehisa et al., 2019). Subsequently, significantly regulated metabolites were mapped to their corresponding metabolic pathways, and Metabolite Set Enrichment Analysis (MSEA) (Xia and Wishart, 2010) was performed. The significance of pathway enrichment was assessed using P-values from the hypergeometric test, enabling the identification of pathways that were significantly enriched under specific experimental conditions.

## 3 Results and discussion

### 3.1 Metabolic overview of strain NSZJ-CX-22 at different cultivation time points

We investigated the endophytic fungus NSZJ-CX-22 (*Arthrinium* sp. E14020a), isolated from the resinous xylem of *A. sinensis*, by inoculating it into a non-living wood chip substrate. Samples were collected on days 7, 14, 21, and 28 for wide-targeted metabolomics (LC-MS/MS) analysis. The experiment comprised 4 time points, with 6 biological replicates per time point. Quality control (QC) confirmed high data reliability: total ion current signals were stable (**Supplementary Figure A**), internal standard retention time deviation was controlled within ±12 s (**Supplementary Figure B**), and QC sample correlation coefficients approached 1 (**Supplementary Figure C**).

**Figure 2:**
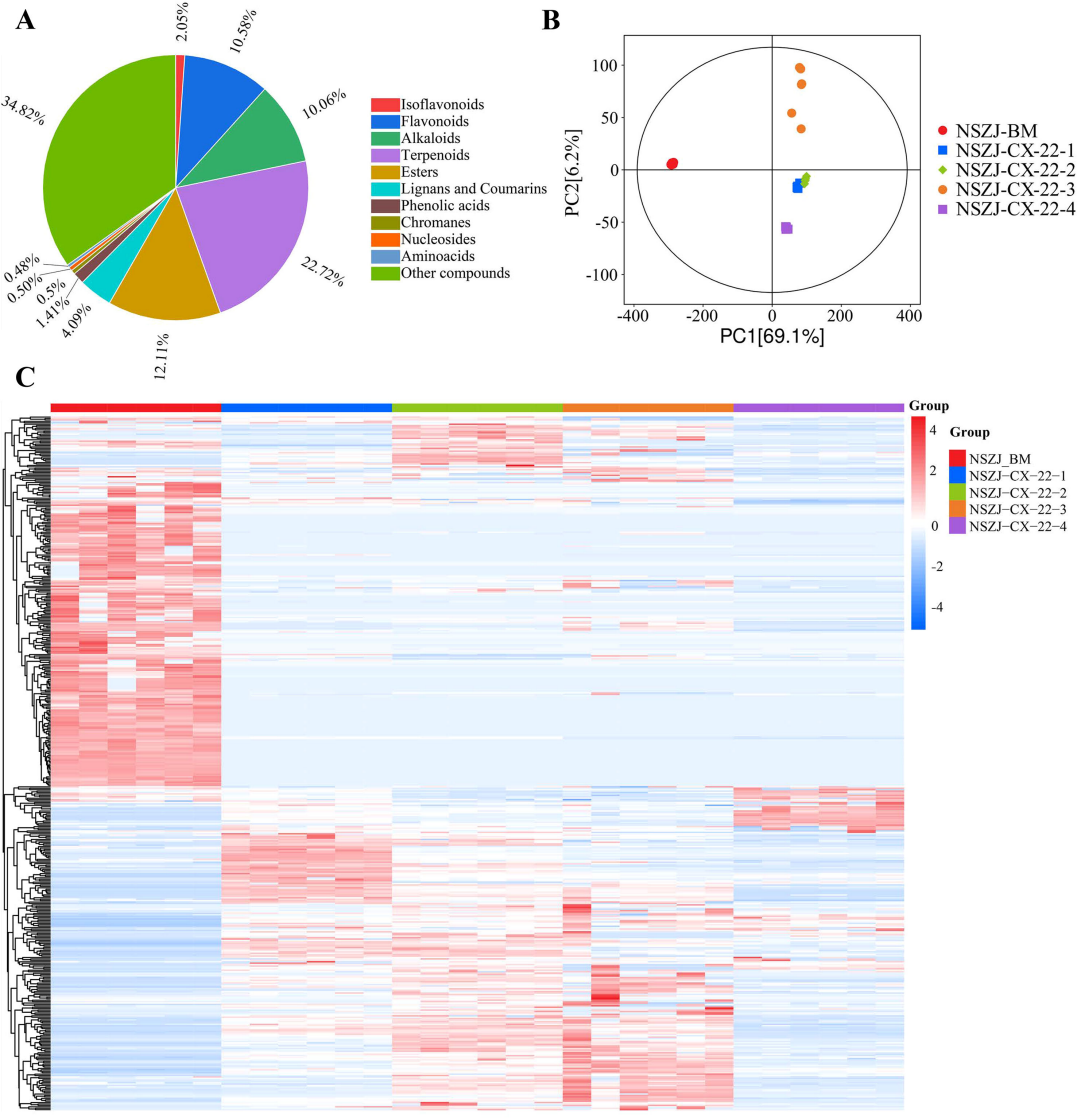
Time-course analysis of the metabolome of endophytic fungi in a medium containing *Aquilaria sinensis* sawdust. **(A)** Statistical classification of metabolites based on UPLC-MS/MS. **(B)** Principal component analysis (PCA). **(C)** Hierarchical clustering heatmap of sesquiterpenoids and chromones.

Metabolic profiling analysis of endophytic fungus-inoculated *A. sinensis* sawdust culture samples at different culture time points was performed using an ultra-performance liquid chromatography-tandem mass spectrometry (UPLC-MS/MS) platform, combined with a self-constructed metabolite database. A total of 14,784 metabolites were identified, covering 11 major categories (**Figure 2A**): terpenoids (3,360 species, including 421 sesquiterpenoids), flavonoids (1,564 species), and alkaloids (1,487 species). Additional classes comprised esters, lignans, coumarins, isoflavonoids, chromones, phenolic acids, nucleosides, amino acids, and others.

Multivariate statistical analysis revealed temporal evolution of the metabolic landscape. High intra-group Pearson correlation coefficients (≈1) confirmed excellent biological reproducibility and reliable differential metabolite identification (**Supplementary Figure D**). Principal component analysis (PCA) showed clear separation between each time point and blank medium (BM) (**Figure 2B**), confirming that the fungal metabolic profiles had undergone significant changes over time. Notably, early time points (7d and 14d) exhibited similar metabolic profiles, whereas later time points (21d and 28d) diverged significantly, indicating “early-phase stability followed by late-phase metabolic flux redistribution”. Hierarchical clustering of sesquiterpenes and chromones further confirmed that inter-group heterogeneity intensified with culture duration, with the most dramatic changes occurring between days 14 and 21 (**Figure 2C**), reflecting significant metabolic network reconfiguration during this period.

At the metabolic regulation level, flux changes in terpenoids, flavonoids, and alkaloids are controlled by key enzymes (CHS, TPS, PKS, P450), while fluctuations in primary metabolites—carbohydrates, amino acid derivatives, and nucleosides—are highly correlated with secondary metabolism timing, suggesting their roles as precursor pools providing carbon/nitrogen skeletons and energy to support time-dependent secondary metabolite biosynthesis in the fungus–substrate system.

### 3.2 Differential analysis of differential metabolites at different culture time points

We conducted targeted comparisons between BM and four key time points (7, 14, 21, and 28 days), identifying 14,555 differential metabolites that delineate the strain’s core metabolic profile in the wood chip substrate. Primary metabolites (4,503 species) were dominated by fatty acids (3,757, 83.4%), followed by amino acids and short peptides (427), carbohydrates and derivatives (141), and nucleosides (69), reflecting efficient utilization of lipid and protein components to build biomass and supply carbon skeletons and energy for secondary metabolism. Among secondary metabolites, terpenoids (3,312), flavonoids (1,558), and alkaloids (1,461) constituted 78.9%, demonstrating robust biosynthetic capacity.

Key metabolites directly related to agarwood characteristic aroma were specifically detected: among 414 sesquiterpenes, 118 agarwood-characteristic skeletons (Eudesmane, Guaiane, Eremophilane, Cadinane, Agarofuran) were identified; among 48 chromones, 2-(2-phenylethyl)chromones and their derivatives were detected. Three core pathways achieve convergence through shared precursors and branch-point enzymes: (i) Shared precursors: Acetyl-CoA is the common carbon source for phenylpropanoid pathways (via phenylalanine) and terpenoid skeleton pathways (MVA/MEP); malonyl-CoA is the common substrate for flavonoid/chromone synthesis. (ii) Branch-point enzymes: High CHS activity during 7–21 days promotes conversion of phenylalanine-derived cinnamic acid to chalcone, providing skeletons for 2-(2-phenylethyl)chromones; TPS activity peaks at day 14, directing FPP toward agarwood-characteristic sesquiterpenes; P450 mediates skeleton modifications, enriching structural diversity. (iii) Regulatory nodes: JA accumulation during 7–14 days, potentially as fungal endogenous or fungal-produced/sensed plant hormone-like signals, upregulates CHS, TPS, and PKS gene expression, synergistically activating core pathways.

**Figure 3:**
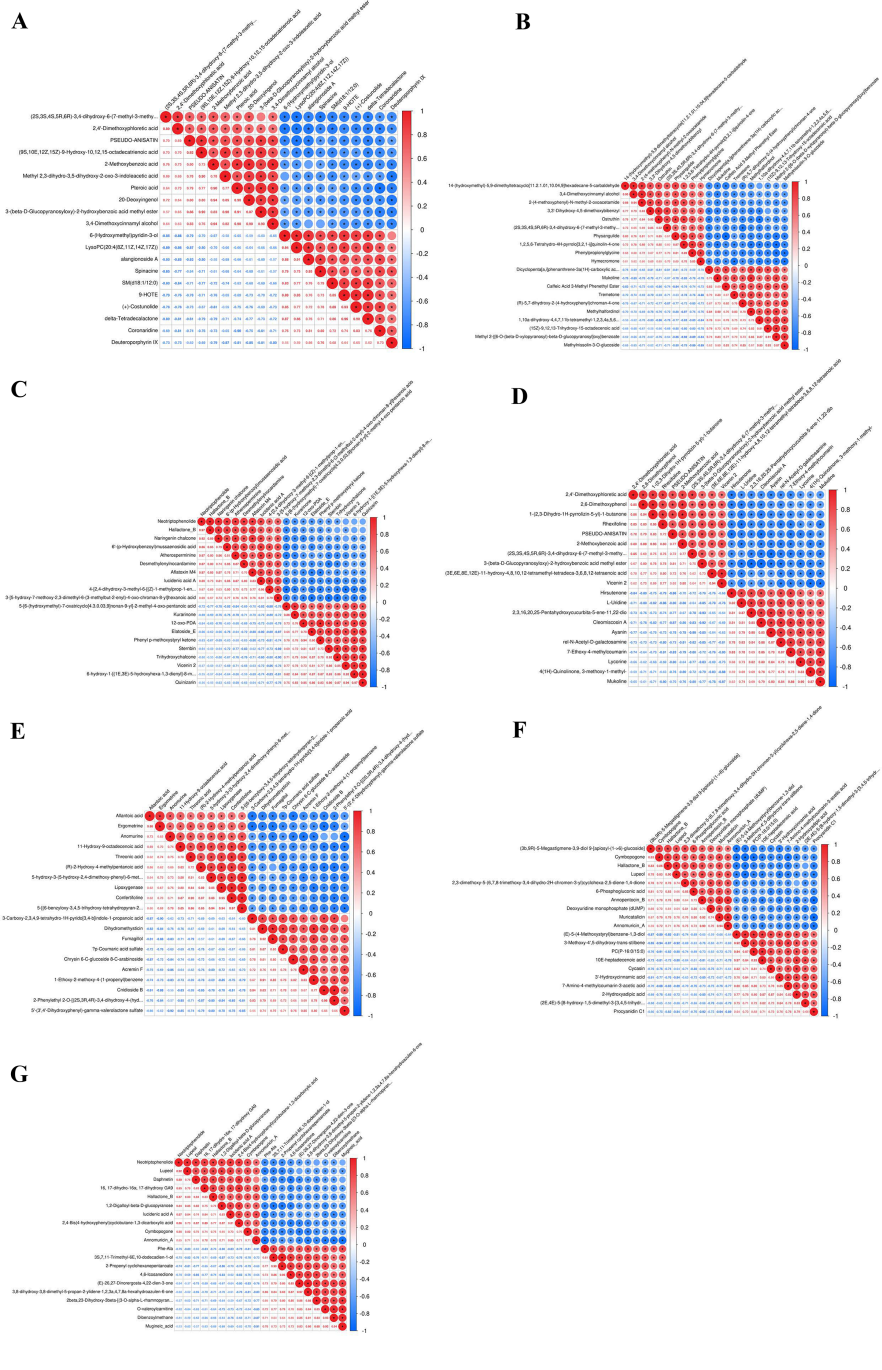
Correlation heatmap of primary and secondary metabolism during endophytic fungus NSZJ-CX-22 cultivation. **(A)** BM *vs* 7d; **(B)** BM *vs* 14d; **(C)** BM *vs* 21d; **(D)** BM *vs* 28d; **(E)** 7d *vs* 14d; **(F)** 14d *vs* 21d; **(G)** 21d *vs* 28d. Red indicates a significant positive correlation, blue indicates a significant negative correlation. The depth of the color represents the strength of the correlation. Arrows indicate the direction of metabolic regulation.

Correlation heatmap analysis revealed significant stage-specific resource trade-offs between primary substrate utilization and secondary metabolite synthesis (**Figure 3**): Early fermentation (BM *vs* 7d(3A)/14d(3B)): Strong negative correlations between primary nutrient utilization and secondary metabolite accumulation. At day 7, fungal-produced terpenoids (PSEUDO-ANISATIN) and phenolic acids showed antagonism with substrate membrane lipids (LysoPC) and alkaloid precursors (r = -0.89), indicating active conversion of primary carbon/nitrogen sources to initiate secondary metabolism. Mid-late fermentation (BM *vs* 21d(3C)/28d(3D)): Metabolic focus shifted among secondary product branches. At day 21, chalcones (Neotriptophenolide) and fungal toxin modules were upregulated with a strong negative correlation to flavonoids (Vicenin 2) and chromone modules (r ≈ -0.80), reflecting metabolic flux redirection within phenylpropanoid downstream branches. Inter-group dynamics: 7d *vs* 14d(3E) showed purine metabolism (Allantoic acid) and lipoxygenase pathway upregulation, antagonizing flavonoid glycosides; 14d *vs* 21d(3F) exhibited a highly positive correlation (r>0.8) between glycolytic intermediates (6-Phosphogluconic acid) and terpenoid (Lupeol) synthesis, with concurrent suppression of stilbenes and proanthocyanidins, marking concentrated carbon flux toward key sesquiterpene skeleton synthesis.

**Figure 4:**
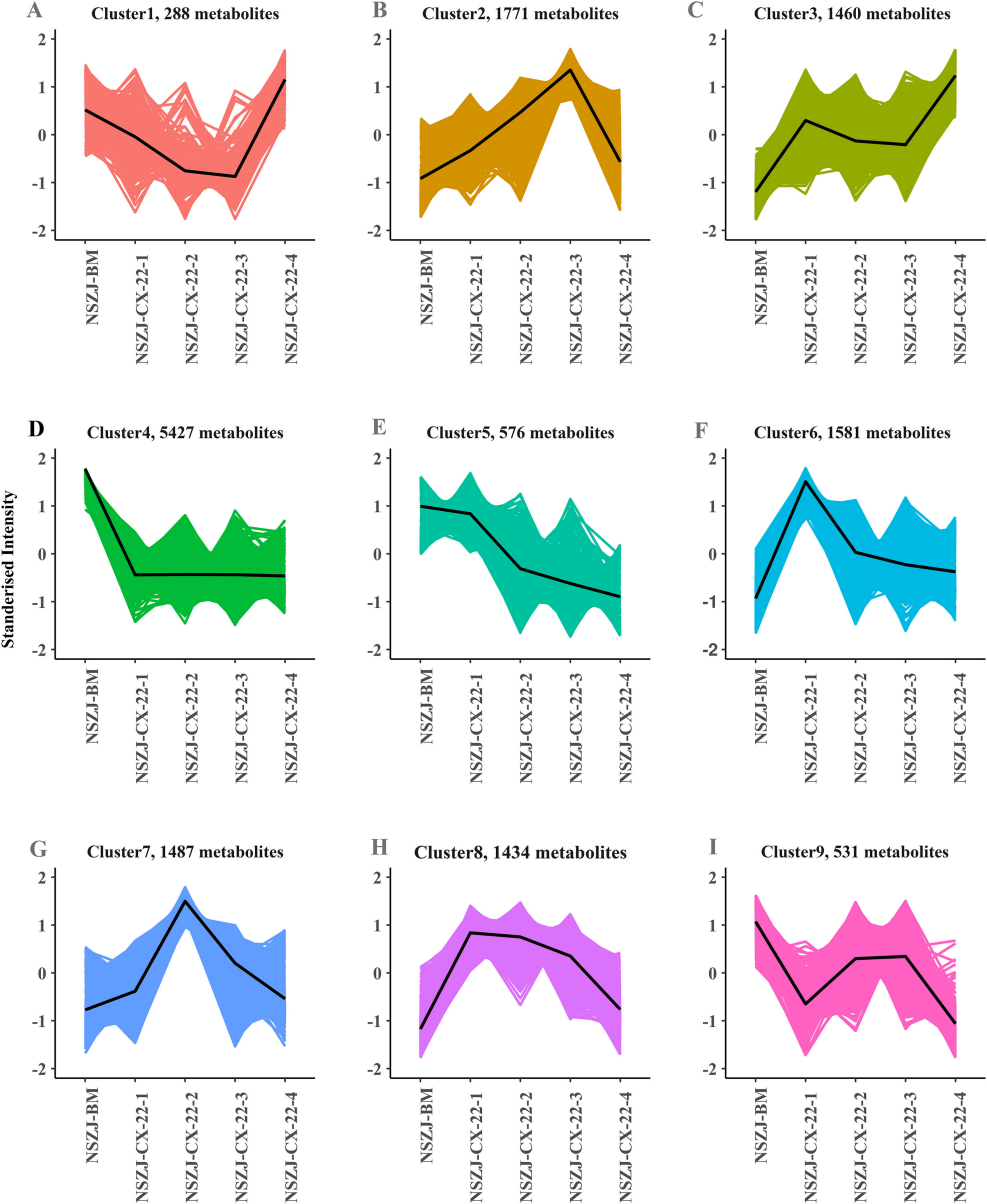
K-means clustering analysis of all differential metabolites. The abscissa represents the sample time-series gradient (culture time points: 0d, 7d, 14d, 21d, 28d). The ordinate indicates the standardised intensity of differential metabolites. **(A–I)** correspond to the nine clusters obtained from the K-means clustering analysis.

K-means clustering analysis divided differential metabolite dynamics into three characteristic phases (**Figure 4**): Phase I (0–7d) Nutrient consumption and precursor accumulation: Clustering results showed that the differential metabolites in clusters 4, 5, and 9 were primarily composed of primary metabolites, such as fatty acids and amino acids, and their contents exhibited a continuous and significant decreasing trend from 0 to 7d (**Table 1**). This characteristic indicated that the strain preferentially initiated primary metabolism at the early stage of cultivation, rapidly accumulating biomass by efficiently degrading and utilising carbon and nitrogen sources in the medium (e.g., fatty acids providing carbon skeletons and amino acids providing nitrogen sources). Meanwhile, it converted primary metabolites into precursors required for secondary metabolism (e.g., acetyl-CoA, malonyl-CoA), thus laying the material and energy foundations for the subsequent onset of secondary metabolism. Notably, this stage was accompanied by the initiation of early-stage sesquiterpenoid synthesis, as evidenced by the accumulation of sesquiterpenoids in clusters 6 and 8, including five characteristic agarwood skeletons (e.g., eudesmane and guaiane), which accumulated significantly during the 0–7d period but gradually degraded after 7d. This dynamic variation suggested that the sesquiterpenoids synthesised in the early stage might act as intermediate metabolites, participating in the reconstruction of more complex secondary metabolites in subsequent stages rather than serving as stably accumulated functional compounds in the end. Phase II (7–21d) Secondary metabolite biosynthetic burst: The sesquiterpenoid biosynthetic pathway was highly activated during this stage. The contents of sesquiterpenoids in cluster 2, especially those with four types of characteristic agarwood skeletons (e.g., eudesmane and eremophilane), peaked at 21d (**Table 2**), indicating that this stage represented the critical period for the strain to synthesise characteristic agarwood sesquiterpenoids. Meanwhile, chromones containing the 2-(2-phenylethyl)chromone skeleton (cluster 8) maintained high levels from 7 to 21d. Despite minor fluctuations, the overall trend of stable accumulation confirmed that the PKS pathway was continuously and highly expressed during this stage, providing enzymatic assurance for the steady synthesis of chromones. Furthermore, the defensive secondary metabolites exhibited a significant upregulation trend, as the contents of alkaloids in cluster 3 and flavonoids in cluster 7 increased continuously from 7 to 21d (**Table 2**). It was hypothesised that the upregulation of such defensive metabolites might be associated with the fungus’s intrinsic stress-adaptation and competitive secondary metabolism under nutrient depletion and metabolite accumulation in the sawdust substrate system (Compton and Ishihara, 2004). Phase III (21–28d) Metabolic restructuring and modification: Clustering results showed that the contents of sesquiterpenoids and chromones in clusters 1, 3, and 7 decreased significantly from 21 to 28d. Combined with the classic mechanisms of fungal metabolic regulation, it was hypothesised that this degradation process might be associated with CYP450 enzyme-mediated oxidative modification or transporter-mediated efflux. CYP450 enzymes can catalyse the hydroxylation and oxidation of compounds such as sesquiterpenoids and chromones, converting them into more active derivatives or metabolites that are prone to excretion. In contrast, transporter-mediated efflux might pump excess accumulated secondary metabolites out of cells, thereby preventing their toxic inhibition on the cells themselves. Meanwhile, another crucial feature of late-phase metabolic regulation was the activation of the phenylpropanoid metabolic hub, as the contents of cinnamic acid and its derivatives in cluster 1 increased significantly from 21 to 28d. As a crucial precursor for lignin synthesis and an important intermediate in the salicylic acid signalling pathway, the accumulation of cinnamic acid suggested a reactivation of the phenylpropanoid hub in the fungus–substrate system. In a non-living sawdust substrate (serving only as a carbon/nitrogen source), this increase more likely reflects fungal metabolic reprogramming toward aromatic precursor supply and downstream derivatization (e.g., for phenolic acids, flavonoid-related metabolites, and chromone-associated scaffolds), rather than host cell-wall reconstruction or host defence signalling. Furthermore, chalcone compounds showed fluctuating changes characterised by the coexistence of synthesis and consumption during this stage, suggesting that the strain might encode CHS. As a critical precursor for the synthesis of flavonoids and chromones, the dynamic metabolic characteristics of chalcones provide an enzymatic basis for the derivatisation and modification of chromone compounds, which further confirms the complexity and flexibility of the secondary metabolic regulation of this strain.

**Table 1:** Statistics on the categories and quantities of secondary metabolites of differential metabolites in Clusters 4, 5 and 9.

Clusters	Amino acids and peptides	Nucleosides	Saccharides	Fatty acids	Chalcones	Cinnamic acids and derivatives
4(5427)	131	12	31	1176	62	97
5(576)	10	1	1	167	6	14
9(533)	16	1	3	45	6	19

**Table 2:** Statistics on the categories and quantities of secondary metabolites of differential metabolites in Clusters 2, 3, 7 and 8.

Clusters	Sesquiterpenes (sesquiterpenes with the characteristic skeleton of agarwood)	Chromones	Flavones	Alkaloids	Chalcones	Cinnamic acids and derivatives
2(1771)	72(22)	10	174	227	16	37
3(1460)	28(4)	2	72	94	10	14
7(1487)	33(13)	4	189	162	28	30
8(1434)	34(8)	10	135	120	13	24

The differences in metabolite composition and variation trends across clusters further revealed the strain’s metabolic regulation. There were significant differences in the types and quantities of differential metabolites between cluster 1 and cluster 6 (**Table 3**). Cluster 1 exhibited a bidirectional variation trend of “decrease from 0d to 21d and increase from 21d to 28d”, which suggested that its regulatory mechanism was closely associated with the adaptation of the strain to different growth phases. During the early cultivation stage (0–21d), the strain was in a competitive phase of rapid growth and a burst in secondary metabolism. The metabolic pathways in cluster 1 may have been inhibited, leading to decreased metabolite levels. After 21d, however, the strain entered the stationary growth phase, exhibiting enhanced environmental adaptability, and the repressed metabolic processes were reactivated, thereby driving an increase in metabolite content. In terms of metabolite composition, fatty acids accounted for the highest proportion in cluster 1, and their content changes dominated the overall variation trend of this cluster. In contrast, cluster 6 showed a trend of “increase from 0d to 7d and decrease from 7d to 28d”, reflecting a dynamic balance between metabolic activation in the early cultivation stage and metabolic inhibition in the later stage. From 0d to 7d, the strain was in a phase of rapid adaptation and growth, with vigorous metabolic activity, leading to a rapid increase in the contents of various metabolites (especially fatty acids, flavonoids, and alkaloids). After 7d, as nutrients in the medium and the accumulation of secondary metabolites, metabolic processes were subjected to feedback inhibition, resulting in a continuous decrease in metabolite content in cluster 6. This variation was also consistent with the overall metabolic phase transition of the strain from “nutrient consumption” to “secondary metabolism” and finally to “metabolic reconstruction”.

**Table 3:** Statistics on the content variation of differential metabolites in Clusters 1 and 6 with culture time.

Clusters	Amino acids and peptides	Nucleosides	Saccharides	Fatty acids	Sesquiterpenes (sesquiterpenes with the characteristic ckeleton of agarwood)	Chromones	Flavones	Alkaloids	Chalcones	Cinnamic acids and derivatives
1(289)	9	1	2	122	11(6)	0	12	17	5	3
6(1581)	29	5	12	658	33(9)	7	116	117	13	19

This study confirmed that strain NSZJ-CX-22 possesses efficient capacity to synthesize agarwood-characteristic aromatic compounds (118 sesquiterpene skeletons and 2-(2-phenylethyl)chromones); the triphasic dynamics closely relate to fungal growth adaptation and self-defensive metabolism; alkaloids may enhance system stress-resistance microenvironment (Muro-Villanueva and Nett, 2023; Xu Yanhong et al., 2016), while cinnamic acid derivatives likely reflect re-activation of aromatic precursor metabolism and late-stage pathway rebalancing in the fungus–substrate system.

### 3.3 Dynamic metabolic characteristics of endophytic fungus NSZJ-CX-22 and accumulation patterns of agarwood-characteristic metabolites at different culture time points

**Figure 5:**
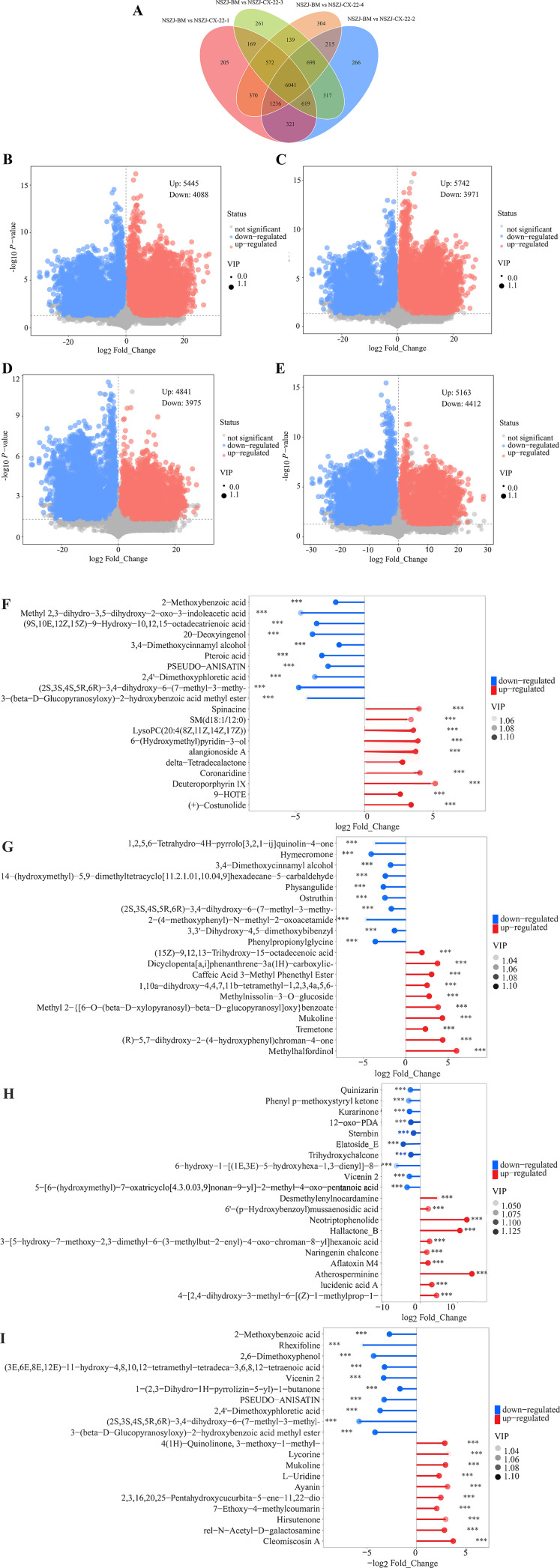
Correlation analysis diagrams of dynamic metabolic characteristics of endophytic fungus NSZJ-CX-22 and accumulation patterns of agarwood-characteristic metabolites at different culture time points. **(A)** Venn diagram showing the overall distribution of differential metabolites and screening results of core differential metabolites in comparisons between each time point and the blank medium (BM). **(B–E)** Volcano plots for statistical analysis of differential metabolites comparing different culture time points (7d, 14d, 21d, 28d) and the blank medium, clearly showing the quantities of upregulated (red) and downregulated (blue) differential metabolites at each time point. **(F–I)** Matchstick plots of the top 10 differential metabolites (up or down) ranked by VIP values in comparisons between the blank medium and each culture time point (7d, 14d, 21d, 28d). Red indicates upregulated compound contents, blue indicates downregulated compound contents, and darker colours of the dots represent higher VIP values. Indicates the significance level, where *p < 0.05, **p < 0.01, and ***p < 0.001.

To elucidate the temporal accumulation patterns of metabolites produced by the endophytic fungus NSZJ-CX-22, we selected four key culture time points (7d, 14d, 21d, and 28d) in this study, with the blank medium (BM) as the control. We established four paired-comparison groups: “BM *vs* 7d, BM *vs* 14d, BM *vs* 21d, and BM *vs* 28d”. Subsequently, we performed metabolomics analysis to characterise differences in metabolites between each culture time point and the blank medium, as well as the variation features of key metabolites. We screened differential metabolites with VIP ≥ 1 in the OPLS-DA model, combined with P < 0.05 from univariate statistical analysis. The results showed that a total of 11,733 differential metabolites were identified in comparisons between each time point and the blank medium, among which 6,041 metabolites exhibited significant variations across all time points (**Figure 5A**). This finding fully demonstrated that the fungus maintained a core metabolic network throughout the entire culture period. **Table 4** provides a detailed list of the total number of differential metabolites, as well as the numbers of upregulated and downregulated metabolites at each time point (**Figures 5B–E**).

**Table 4:** Statistics on the total number, up-regulation and down-regulation of differential metabolites at each culture time point.

Groups	Total number of differential metabolites	Up	Down
BM *vs* 7d	9533	5445	4088
BM *vs* 14d	9713	5742	3971
BM *vs* 21d	8816	4841	3975
BM *vs* 28d	9575	5163	4412

The time point with the greatest increase in differential metabolites was 14d, consistent with the results of the culture time trend analysis presented in Section 3.2. The number of differential metabolites on the 21d decreased significantly compared with that on the 14d. Although the number of downregulated metabolites remained consistent with the earlier stage, the number of upregulated differential metabolites dropped sharply, suggesting that the strain’s precursors for metabolite synthesis were extensively consumed, making it difficult to sustain efficient metabolic activity. On the 28d, the number of upregulated metabolites showed a slight recovery but remained significantly lower than on the 14th day, indicating that the strains’ growth and reproductive vigour had already declined. Although the strain could synthesise energy-related metabolites, such as fatty acids, it was unable to restore the highly efficient metabolic activity observed in the earlier stage.

An in-depth analysis of the culture time-dependent variation data for primary metabolites, presented in **Table 5**, revealed that the overall upregulation and downregulation of primary metabolites remained stable across the four key time points (7d, 14d, 21d, and 28d). As mentioned above, this indicated that the culture system could continuously meet the strains’ basic requirements for primary metabolites throughout the 28-day cultivation period. Specifically, the synthesis of amino acids and oligopeptides remained stable for the first 14 days, decreased sharply by the 21d, and showed a slight recovery by the 28d. However, it did not return to the initial level. It implied that the strain had a high demand for amino acids and oligopeptides, and its synthetic capacity declined during the period from 14 to 28d, indicating that synthesis had reached its peak and was starting to decline. The levels of nucleosides and carbohydrates reached their lowest point on the 21d, indicating that primary metabolism had entered a stagnant phase at that time. The initial fatty acid upregulation rate was relatively slow; however, their content remained high from 7 to 28d. This might indicate that the strain had adjusted its metabolic energy allocation strategy, converting short-acting energy sources (amino acids and carbohydrates) into long-acting energy reserves (fatty acids) for storage and reuse, thereby coping with the continuous consumption of medium substrates and growth challenges under adverse environmental conditions.

**Table 5:** Statistics on the variation of the number of up-regulated and down-regulated primary metabolites at each culture time point.

Compounds	Change trend	BM *vs* 7d	BM *vs* 14d	BM *vs* 21d	BM *vs* 28d
Amino acids and peptides	Up	206	201	132	180
	Down	102	98	98	104
Nucleosides	Up	43	38	33	46
	Down	8	9	7	7
Saccharides	Up	57	60	41	57
	Down	24	21	26	24
Fatty acids	Up	1378	1673	1554	1614
	Down	863	843	855	876

In-depth analysis based on the culture time-dependent variation data of secondary metabolites presented in **Table 6** revealed that sesquiterpenoids (including those with agarwood-characteristic skeletons) exhibited an overall trend of “increase–decrease–recovery”. Specifically, 156 upregulated sesquiterpenoids were identified in both the “BM *vs* 7d” and “BM *vs* 14d” comparisons, among which 34 and 44 compounds possessed agarwood-characteristic skeletons, respectively, indicating vigorous synthesis activity in the early stage. In contrast, the number of upregulated sesquiterpenoids decreased to 120 (31 with agarwood-characteristic skeletons) and 124 (37 with agarwood-characteristic skeletons) in the “BM *vs* 21d” and “BM *vs* 28d” comparisons, respectively, suggesting a decline in the sesquiterpenoid biosynthetic capacity of the strain. As the dominant group of secondary metabolites, flavonoids also displayed active synthesis in the early stage, with 526 and 525 upregulated compounds detected in the “BM *vs* 7d” and “BM *vs* 14d” comparisons, respectively. Although their biosynthetic capacity weakened in the later stage, the total amount of flavonoids remained high. Among other secondary metabolites, except for chromones whose abundance decreased on the 21d, chalcones, cinnamic acid and its derivatives, and alkaloids all exhibited a trend of “active synthesis in the early stage, followed by weakened but stably maintained synthesis in the later stage”. This phenomenon indicated that the strain had adapted to the growth environment and entered a phase of stable metabolic activity. However, the decreased accumulation of primary metabolites might imply the termination of this stable metabolic phase.

**Table 6:** Statistics on the variation of the number of up-regulated and down-regulated secondary metabolites at each culture time point.

Compounds	Change trend	BM *vs* 7d	BM *vs* 14d	BM *vs* 21d	BM *vs* 28d
Sesquiterpenes (sesquiterpenes with the characteristic skeleton of agarwood)	Up	156(43)	156(44)	120(31)	124(37)
	Down	131(32)	120(30)	113(29)	142(34)
Flavones	Up	526	525	451	474
	Down	534	521	525	591
Chalcones	Up	62	58	44	52
	Down	46	51	46	52
Cinnamic acids and derivatives	Up	92	99	74	79
	Down	88	84	78	99
Chromones	Up	24	23	19	22
	Down	9	8	7	10
Alkaloids	Up	539	535	449	477
	Down	433	424	430	476

The variation trend of secondary metabolites is closely correlated with the stability of the primary metabolic system. Primary metabolism continuously supplies energy to support secondary metabolism; however, as a direct energy source, the strains’ energy utilisation efficiency decreases, resulting in a slowdown in metabolite synthesis on the 21d of culture. Meanwhile, the synthesis, transformation, and utilisation of fatty acids drive a slight rebound in secondary metabolite synthesis on 28d. Based on these findings, precise control of culture duration is required for the directional accumulation of characteristic sesquiterpenes and chromones in agarwood. Sesquiterpenes should be harvested at an early stage of culture when their synthesis is active, whereas chromones should be obtained while avoiding the energy metabolism nadir around the 21d.

Multivariate statistical analysis was performed to identify the top 10 differential metabolites ranked by VIP (top 3), revealing the metabolic regulatory characteristics at different culture stages. For the “BM *vs* 7d” comparison (**Figure 5F**), the significantly downregulated metabolites included 3,4-dimethoxycinnamyl alcohol (a phenolic acid derivative potentially involved in lignin biosynthesis), pteroic acid (folic acid), and 2-methoxybenzoic acid (organic acid). In contrast, the metabolites that were upregulated were (+)-costunolide (a sesquiterpene lactone), delta-tetradecalactone (a macrolactone), and 9-HOTE (fatty acid). The upregulation of these metabolites suggested the enhanced activity of terpenoid and PKS biosynthetic pathways. For the “BM *vs* 14d” comparison (**Figure 5G**), the downregulated metabolites were 3,3’-dihydroxy-4,5-dimethoxybibenzyl, 14-(hydroxymethyl)-5,9-dimethyltetracyclo[11.2.1.0¹,¹^0^.0^4^,^9^]hexadecane-5-carbaldehyde (a complex terpenoid possibly associated with nutrient depletion), and phenylpropionylglycine (amino acid derivatives, pharmaceutical synthesis intermediates). The upregulated metabolites included methylnissolin-3-O-glucoside (a flavonoid derivative), tremetone (a neurotoxic benzofuran derivative indicative of potential biological defence), and (15Z)-9,12,13-trihydroxy-15-octadecenoic acid (a fatty acid). For the “BM *vs* 21d” comparison (**Figure 5H**), the downregulated metabolites were sternbin (a chromone derivative whose decrease might be attributed to reduced enzymatic synthesis or accelerated degradation), trihydroxychalcone (chalcone), and kurarinone (flavonoids). The upregulated metabolites were atherosperminine (an alkaloid with antitumor activity, suggesting the strengthened defensive function of secondary metabolism), neotriptophenolide (a sesquiterpene lactone with an α,β-unsaturated ketone moiety, potentially synthesised efficiently via the MVA pathway), and hallactone B (lactones). For the “BM *vs* 28d” comparison (**Figure 5I**), the downregulated metabolites included 1-(2,3-dihydro-1H-pyrrolizin-5-yl)-1-butanone (a pyrrolizidine alkaloid precursor, possibly suppressed due to pathway inhibition), (3E,6E,8E,12E)-11-hydroxy-4,8,10,12-tetramethyl-tetradeca-3,6,8,12-tetraenoic acid (a polyunsaturated fatty acid whose decrease might indicate oxidative decomposition), and 2-methoxybenzoic acid (consistently downregulated as observed in the 7 d group, serving as a stable biomarker). The upregulated metabolites were cleomiscosin A (an anticoagulant coumarin derivative potentially synthesised via the phenylpropanoid pathway), 4(1H)-quinolinone, 3-methoxy-1-methyl- (a quinoline alkaloid possibly derived from amino acid metabolic reconstruction), and L-uridine (nucleoside).

In summary, this study clarified the dynamic metabolic variation patterns of the endophytic fungus NSZJ-CX-22 over a 7d–28d culture period via metabolomics analysis: the strain maintained the stability of its core metabolic network overall, with the 14d representing the peak of active differential metabolite synthesis, the 21d marking the entry into a metabolic stagnant phase, and a slight recovery in metabolic activity observed on the 28d without returning to the earlier high-efficiency level. At the primary metabolism level, short-acting energy substrates such as amino acids and nucleosides showed an initial period of stability followed by decline. In contrast, fatty acids, as long-acting energy sources, maintained high, sustained expression levels, reflecting the strains’ adaptive adjustment of energy allocation. At the secondary metabolism level, core products, including sesquiterpenoids (characterised by agarwood skeletons) and flavonoids, displayed active synthesis in the early stage, with a weakened but overall stable synthetic capacity in the later stage; specific products, such as chromones, showed stage-specific fluctuations. Analysis of the top 10 differential metabolites ranked by VIP values revealed the regulatory characteristics of crucial substances (e.g., terpenoids, polyketides, and alkaloids) across different culture stages, clarifying the intrinsic correlation that energy supply from primary metabolism underpins the dynamic changes in secondary metabolism. The findings of this study provide evidence for the targeted accumulation of objective products. Sesquiterpenoids are preferable to harvest during the early culture stage, while chromone production should be avoided during the energy metabolism nadir around the 21d. These results provide a theoretical foundation for optimising strain culture conditions and enhancing the yield of target metabolites.

Process window and harvesting strategy: Based on “day-14 metabolic peak, day-21 energy trough, day-28 modification surge,” we recommend: (i) Sesquiterpene collection during 7–14d window (TPS activity peak + FPP targeting); (ii) Chromone collection before day 14 or after day 28, avoiding day-21 energy deficit; (iii) For chromone derivatives, leverage day 21–28 P450-mediated modifications to enhance structural diversity and aroma complexity. This temporal program provides executable process references for segmented fermentation harvesting and flux control.

### 3.4 Metabolic dynamics variations and key differential metabolites of endophytic fungus NSZJ-CX-22 at adjacent culture time points

**Figure 6:**
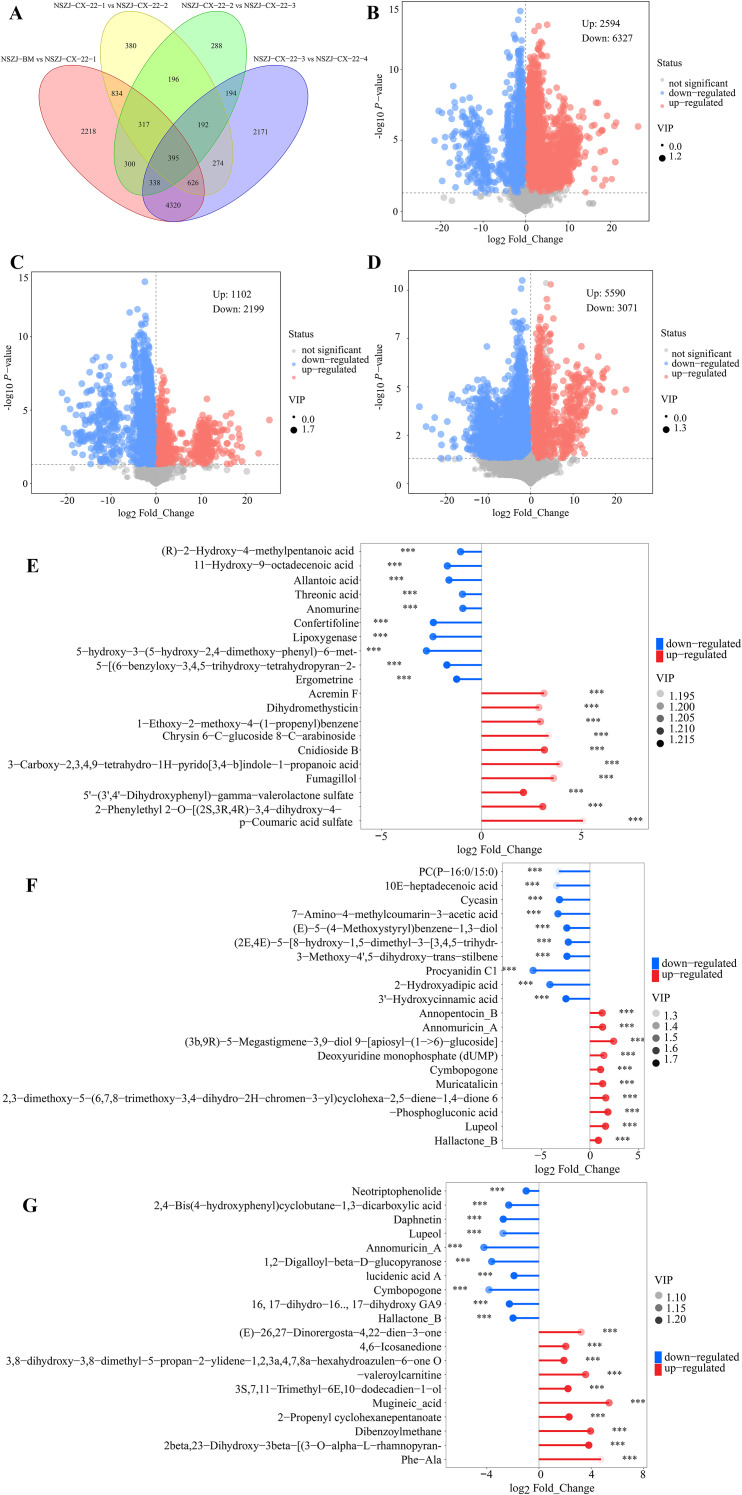
Correlation analysis diagrams of metabolic dynamic variations and key differential metabolites of endophytic fungus NSZJ-CX-22 at adjacent culture time points. **(A)** Venn diagram showing the overall distribution of differential metabolites and the screening results for core differential metabolites in comparisons between adjacent culture time points. **(B–D)** Volcano plots for statistical analysis of differential metabolites in comparisons between adjacent culture time points (7d *vs* 14d, 14d *vs* 21d, 21d *vs* 28d), which clearly show the quantities of upregulated (red) and downregulated (blue) differential metabolites at each pair of adjacent time points. **(E–G)** Matchstick plots of the top 10 differential metabolites (up and down) ranked by VIP values in comparisons between adjacent culture time points (7d *vs* 14d, 14d *vs* 21d, 21d *vs* 28d). Red indicates upregulated compound contents, blue indicates downregulated compound contents, and darker colours of the dots represent higher VIP values. Indicates the significance level, where *p < 0.05, **p < 0.01, and ***p < 0.001.

To elucidate the continuous variation in the metabolic characteristics of the endophytic fungus NSZJ-CX-22, four key culture time points (7th, 14th, 21st, and 28th day) were selected in this study, with the blank medium (BM) serving as the control. Four paired comparison groups, namely “BM *vs* 7d, 7d *vs* 14d, 14d *vs* 21d, and 21d *vs* 28d”, were established. Metabolomics analysis was performed to characterise metabolite differences between adjacent culture stages and to identify the dynamic variation in crucial metabolites. Differential metabolites were screened and identified based on VIP ≥ 1 in the OPLS-DA model and P < 0.005 from univariate statistical analysis. The results showed that 13,043 differential metabolites were identified in all pairwise comparisons of consecutive time points, among which 395 metabolites exhibited significant variations across all stages (**Figure 6A**). This finding indicated that culture duration is a crucial factor regulating the metabolic activity of this endophytic fungus. A set of core metabolites is continuously regulated throughout the culture period. Among these persistently varying metabolites, 10 were significantly upregulated, and 34 were significantly downregulated (**Table 7**), suggesting that the strains’ metabolic network exhibits a specific regulatory trend during long-term cultivation.

**Table 7:** Shared differential metabolites in response to culture time point: 10 significantly up-regulated and 34 significantly down-regulated, indicating that cultivation duration markedly influences the metabolic activity of the endophytic fungus.

No.	Compounds	CAS	Class	Changing trend
1	(1xi,4xi,6xi)-Carvone_oxide	–	Terpenoids	Up
2	Epinepetalactone	17257-15-7	Terpenoids	Up
3	Isomintlactone	–	Terpenoids	Up
4	Limonene aldehyde	–	Terpenoids	Up
5	Perilla_ketone	553-84-4	Terpenoids	Up
6	Myrtenyl methyl ether	–	Terpenoids	Up
7	16beta-16-Hydroxy-3-oxo-1,12-oleanadien-28-oic acid	–	Terpenoids	Up
8	Benzyl Isothiocyanate	622-78-6	Aromatic derivatives	Up
9	2-Butyl-4-ethyl-5-methyloxazole	–	Others	Up
10	Adenosine monophosphate (AMP)	61-19-8	Others	Up
11	Jaranol	3301-49-3	Shikimates and phenylpropanoids	Down
12	Flavone base + 3O, 2MeO, O-Hex-dHex	53766-40-8	Shikimates and phenylpropanoids	Down
13	Dihydroresveratrol 3-O-glucoside	–	Shikimates and Phenylpropanoids	Down
14	Epimedin_K	–	Shikimates and phenylpropanoids	Down
15	Hydroxytyrosol Acetate	69039-02-7	Shikimates and phenylpropanoids	Down
16	Echoside C	–	Shikimates and phenylpropanoids	Down
17	Rhamnazin_3-rutinoside	64527-08-8	Shikimates and phenylpropanoids	Down
18	5-Hydroxy-3-[4-hydroxy-2-[(2S,3R,4S,5S,6R)-3,4,5-trihydroxy-6-(hydroxymethyl)tetrahydropyran-2-yl]oxy-phenyl]-7-methoxy-chromen-4-one	–	Shikimates and phenylpropanoids	Down
19	2-Hydroxyacetophenone	–	Shikimates and phenylpropanoids	Down
20	Velutin	25739-41-7	Shikimates and phenylpropanoids	Down
21	(2S,3R,4S,5S,6R)-2-(4-allyl-2-methoxy-phenoxy)-6-[[(2R,3R,4R)-3,4-dihydroxy-4-(hydroxymethyl)tetrahydrofuran-2-yl]oxymethyl]tetrahydropyran-3,4,5-triol	136083-96-0	Shikimates and phenylpropanoids	Down
22	3,3,5-Trihydroxy-2-(4-hydroxy-3-methoxyphenyl)-2,7-dimethoxychromen-4-one	–	Shikimates and phenylpropanoids	Down
23	(-)-Syringaresinol	6216-81-5	Shikimates and phenylpropanoids	Down
24	(11S,13)-Dihydro-8-deoxylactucin	–	Terpenoids	Down
25	5,9b-Dihydroxy-6,6,9a-trimethyl-5,5a,8,9-tetrahydro-3H-benzo[g]isobenzofuran-1,7-dione	–	Terpenoids	Down
26	Glaucarubolone_15-O-beta-D-glucopyranoside	89202-76-6	Terpenoids	Down
27	(1S,4aS,7S,7aS)-1-[(2S,3R,4S,5S,6R)-6-[[(E)-3-(3,4-dihydroxyphenyl)prop-2-enoyl]oxymethyl]-3,4,5-trihydroxy-tetrahydropyran-2-yl]oxy-7-hydroxy-7-methyl-4a,5,6,7a-tetrahydro-1H-cyclopenta[c]pyran-4-carboxylic acid	–	Terpenoids	Down
28	Loganin	18524-94-2	Terpenoids	Down
29	26-Glucosyl-1,3,11,22-tetrahydroxyergosta-5,24-dien-26-oate	–	Terpenoids	Down
30	Labriformidin	–	Terpenoids	Down
31	2-Propenoic acid, 3-(4-hydroxy-3-methoxyphenyl)-, (1aS,1bS,2S,5aR,6S,6aS)-2-(beta-D-glucopyranosyloxy)-1a,1b,2,5a,6,6a-hexahydro-1a-(hydroxymethyl)oxireno[4,5]cyclopenta[1,2-c]pyran-6-yl ester, (2E)-	–	Terpenoids	Down
32	Cichorioside L	–	Terpenoids	Down
33	Sylvestroside I	–	Terpenoids	Down
34	2-(9-Hydroxy-2,6,9a-trimethyl-4-oxo-6-vinyl-3,5,5a,7,8,9-hexahydro-2H-pyrano[2,3-b]chromen-7-yl)-2-methyl-propanoic acid	–	Terpenoids	Down
35	[(6E,10Z)-6-formyl-5-hydroxy-10-(hydroxymethyl)-3-methylene-2-oxo-3a,4,5,8,9,11a-hexahydrocyclodecafuran-4-yl] 2-methylbutanoate	72023-19-9	Terpenoids	Down
36	Alangimarckine	–	Alkaloids	Down
37	17-O-acetylnorajmaline	–	Alkaloids	Down
38	Isocorynoxeine	51014-29-0	Alkaloids	Down
39	N-Formyldemecolcine	–	Alkaloids	Down
40	5-(3’,4’-Dihydroxyphenyl)-gamma-valerolactone	–	Others	Down
41	4-Methoxyindol-3-ylmethyl glucosinolate	–	Others	Down
42	Neocryptotanshinone	109664-02-0	Polyketides	Down
43	[4,5-Dihydroxy-6-(hydroxymethyl)-2-[2,4,6-trihydroxy-3-(4-hydroxybenzoyl)phenyl]tetrahydropyran-3-yl] 3,4,5-trihydroxybenzoate	–	Polyketides	Down
44	3-Methyl-2,4-nonanedione	–	Fatty acids	Down

**Table 8** further illustrates the quantitative variation characteristics of differential metabolites. The period from 0d to 7d represented the most metabolically active stage (**Figure 6B**), during which the numbers of synthesised and degraded compounds were basically balanced, reflecting vigorous processes of substance synthesis and transformation in the strain at the initial stage of cultivation. After entering the 7d to 21d stage (**Figures 6B, C**), both the number of upregulated synthesized compounds and the number of degraded compounds decreased continuously, indicating a significant reduction in the overall metabolic activity of the culture system during this period. Three main reasons were hypothesised to account for this phenomenon. First, substantial consumption of nutrient substrates such as carbon and nitrogen sources in the medium compromised the sustainability of subsequent metabolic reactions, thereby inducing a nutrient limitation effect. Second, the activity of key enzymes in synthetic pathways was subjected to feedback inhibition by metabolic intermediates, or deviations in system pH from the optimal range during cultivation led to conformational changes in enzymes, thereby decreasing the rate of synthetic reactions. Third, with the increasingly dense growth of strain mycelia and the continuous accumulation of metabolites, the mass transfer efficiency of the culture system declined, hindering oxidative phosphorylation in the strain, reducing ATP production, and ultimately exerting significant inhibition on energy-dependent synthetic pathways. In the 21d to 28d stage (**Figure 6D**), however, the metabolic level of the culture system exhibited a marked recovery, characterised by a rapid increase in the number of upregulated synthesised metabolites as well as a synchronous rise in the number of downregulated degraded compounds. The driving factors underlying this metabolic resuscitation phenomenon could be summarised into three aspects. First, nutrient-deficient conditions triggered the substrate-reutilization mechanism. After the exhaustion of readily utilisable carbon sources (e.g., carbohydrates) in the early stage, fatty acids were mobilised and decomposed for energy supply. Meanwhile, increased protein degradation released amino acids that could be recycled as nitrogen sources, and the nitrogen assimilation pathway was subsequently reactivated to support the biosynthesis of nucleotide sugars. Second, the massive consumption and metabolite synthesis during the early cultivation stage caused fluctuations in system pH. In this stage, the strain had gradually adapted to the environment, and the system pH tended to stabilise, which was more conducive to enzymatic reactions and cell growth; third, nutrient stress acted as an environmental signal to activate the stress response mechanism of the strain, leading to the specific induction of secondary metabolic pathways. The strain enhanced its environmental adaptability by synthesising a large quantity of bioactive metabolites.

**Table 8:** Statistics on the total number, up-regulation and down-regulation of differential metabolites between adjacent culture time points.

Groups	Total number of differential metabolites	Up	Down
BM *vs* 7d	9533	5445	4088
7d *vs* 14d	8921	2594	6327
14d *vs* 21d	3201	1102	2199
21d *vs* 28d	8661	5590	3071

To further elucidate the functional implications of metabolite variations, this study analysed the primary metabolic characteristics across the four groups of adjacent time points by integrating the variation trends of primary metabolites presented in **Table 9**. The results demonstrated that the content of different categories of primary metabolites showed distinct upward or downward trends at each time interval, with considerable variation in the magnitude of changes among various substances. This indicated that primary metabolites were consistently regulated in a dynamically balanced regulation throughout the strain’s cultivation period. For amino acids and oligopeptides, the number of upregulated compounds was significantly higher than that of downregulated ones during the initial cultivation stage (0d–7d), suggesting that the strain could rapidly utilise nitrogen sources in the medium and synthesise abundant amino acids and oligopeptides to meet the demands of rapid mycelial growth and metabolism. During the mid-cultivation stage (7d–21d), the number of upregulated and downregulated compounds in this class tended to balance, suggesting that fungal metabolism entered a relatively stable phase and that growth might have progressed into the stationary phase. In the late cultivation stage (21d–28d), the number of upregulated compounds again significantly exceeded that of downregulated ones. This phenomenon might be attributed to the activation of fungal autophagy mechanisms under nutrient deprivation, in which the breakdown of endogenous structural proteins or the redirection of metabolic flux led to the accumulation of amino acids and oligopeptides. Nucleosides exhibited relatively small overall variation, with mild fluctuations in the number of upregulated and downregulated compounds across all stages, indicating their relative stability during the cultivation of the strain. This characteristic is closely associated with the biological functions of nucleosides, as core substances for genetic information transmission and energy metabolism; their synthesis and consumption rates must be maintained at a relatively constant level throughout the fungal growth cycle to ensure the normal operation of basic life activities. Regarding carbohydrates, more upregulated compounds were observed during the initial stage (0d–7d), which is presumed to result from the rapid absorption of carbon sources in the medium by the fungus and the synthesis of polysaccharides as energy reserves. During the mid-stage (7d–14d), the number of downregulated compounds exceeded that of upregulated ones, indicating that the fungus had entered a phase of rapid growth and was extensively consuming stored carbohydrates for energy supply. In the late stage (14d–28d), the number of upregulated carbohydrates recovered, possibly because the fungus resynthesized or accumulated specific carbohydrates by regulating glycolytic pathways (e.g., gluconeogenesis) under nutrient limitation stress, thereby enhancing its osmotic regulation capacity. For fatty acids, the number of upregulated compounds was significantly higher than that of downregulated ones in the initial stage (0d–7d), indicating that the fungus required massive fatty acid synthesis to build cellular structures such as cell membranes during the phase of rapid growth and proliferation. Although the number of upregulated and downregulated fatty acids fluctuated during the mid and late stages, the overall trend remained dominated by upregulation. This might be explained by the fungus continuously adjusting the fatty acid composition and fluidity of cell membranes to adapt to environmental changes, while simultaneously maintaining a dynamic balance between fatty acid storage and catabolic metabolism.

**Table 9:** Statistics on the variation of the number of up-regulated and down-regulated primary metabolites between adjacent culture time points.

Compounds	Change trend	BM *vs* 7d	7d *vs* 14d	14d *vs* 21d	21d *vs* 28d
Amino acids and Peptides	Up	206	76	47	159
	Down	102	152	66	69
Nucleosides	Up	43	22	12	17
	Down	8	15	9	14
Saccharides	Up	57	21	21	41
	Down	24	39	12	13
Fatty acids	Up	1378	281	128	1263
	Down	863	1536	394	372

An integrated analysis of the variation trends in secondary metabolites presented in **Table 10** revealed that the secondary metabolic characteristics also exhibited distinct stage-specific differences across different cultivation phases. During the early cultivation stage (0d–7d), the numbers of both upregulated and downregulated compounds across multiple categories (including sesquiterpenoids, flavonoids, and alkaloids) were relatively high. This indicated that secondary metabolic reactions were intense in this stage, with vigorous synthesis and transformation of various compounds, consistent with the strain’s stress-induced metabolic characteristics as it adapted to the new culture environment. At the 7d to 14d stage, the numbers of upregulated and downregulated compounds for certain classes (e.g., flavonoids and alkaloids) reached their peaks. It was hypothesised that this stage involved critical processes driving compound degradation or their conversion toward other metabolic branches. Notably, JA content, a plant hormone, increased significantly during this period. This phenomenon suggested that the endophytic fungus might enhance its environmental adaptability by the significant increase of jasmonate-related metabolites (e.g., JA and derivatives) coincided with shifts in secondary metabolism, suggesting that jasmonate-like chemistry may be involved in the fungus’s internal regulatory network or stress-associated metabolic programming in the substrate culture system (Chen et al., 2017; Lv et al., 2019; Okudera and Ito, 2009; Schulz et al., 2002; Zhang et al., 2022a, Zhang et al., 2022b). In the 14d to 21d stage, the number of upregulated and downregulated compounds across all categories decreased, and secondary metabolism tended to stabilise. Among these metabolites, dihydrojasmonic acid increased significantly. Given that dihydrojasmonic acid has a more stable chemical structure than jasmonic acid, this change implied that metabolic activity transitioned from an intense stress response in the early stage to a more moderate and homeostatic regulatory state. During the late cultivation stage (21d–28d), the number of upregulated compounds (including sesquiterpenoids, flavonoids, and alkaloids) increased again. It was inferred that novel environmental or physiological factors drove the synthesis of secondary metabolites during this period. Meanwhile, the content of methyl dihydrojasmonate increased significantly. Given its high volatility and aromatic properties, this result demonstrated that the strain’s metabolic focus gradually shifted toward the biosynthesis of secondary metabolites and aroma-related compounds in the late stage, which might represent an important survival strategy for the strain in response to nutrient stress.

**Table 10:** Statistics on the variation of the number of up-regulated and down-regulated secondary metabolites between adjacent culture time points.

Compounds	Change trend	BM *vs* 7d	7d *vs* 14d	14d *vs* 21d	21d *vs* 28d
Sesquiterpenes (sesquiterpenes with the characteristic skeleton of agarwood)	Up	156(43)	88(24)	44(8)	166(46)
	Down	131(32)	190(55)	58(18)	104(28)
Flavones	Up	526	339	109	729
	Down	534	805	285	414
Chalcones	Up	62	40	18	65
	Down	46	70	28	40
Cinnamic acids and derivatives	Up	92	61	14	97
	Down	88	116	48	65
Chromones	Up	24	6	0	15
	Down	9	19	10	12
Alkaloids	Up	539	319	138	590
	Down	433	649	246	432

Multivariate statistical analysis was performed to identify the top 10 differential metabolites ranked by VIP (top 3), revealing the core metabolic regulatory characteristics across different culture stages. The attributes of differential metabolites in the “BM *vs* 7d” comparison have been elaborated in Section 3.3. For the “7d *vs* 14d” comparison (**Figure 6E**), the significantly downregulated metabolites included ergometrine (an alkaloid that is abundantly synthesized by the fungus in the initial culture stage for defensive mechanisms; its synthesis may decrease with prolonged cultivation due to feedback inhibition or resource allocation adjustment), 5-[(6-benzyloxy-3,4,5-trihydroxy-tetrahydropyran-2-yl)methoxy]-3-hydroxy-3-methyl-5-oxo-pentanoic acid (an organic acid that may be massively synthesized in the early stage as energy reserves or structural components, while the metabolic flux shifts toward more efficient pathways in the mid-stage), and confertifoline (terpenoids). The upregulated metabolites were 5’-(3’,4’-dihydroxyphenyl)-gamma-valerolactone sulfate (a lactone whose upregulation may be associated with the enhanced secondary metabolic activity of the endophytic fungus), cnidioside B (glycosides), and 2-phenylethyl 2-O-[(2S,3R,4R)-3,4-dihydroxy-4-(hydroxymethyl)tetrahydro-2-furanyl]-beta-D-glucopyranoside (a glycoside whose upregulation may be linked to the adjustment of carbohydrate metabolic pathways, serving to supply energy or synthesize biomolecules). For the “14d *vs* 21d” comparison (**Figure 6F**), the downregulated metabolites were 3’-hydroxycinnamic acid (phenolic acids), 3-methoxy-4’,5-dihydroxy-trans-stilbene (phenols), and (E)-5-(4-methoxystyryl)benzene-1,3-diol (phenolic acids that may be abundantly synthesized in the mid-culture stage to scavenge free radicals and protect cells from oxidative damage, and their levels decrease in the late stage as oxidative stress diminishes). The upregulated metabolites included hallactone B (its upregulation may be related to the activation of specific metabolic pathways in the endophytic fungus, potentially for synthesizing bioactive compounds such as antimicrobial agents), 6-phosphogluconic acid (an intermediate product of the pentose phosphate pathway; its upregulation indicates that this pathway may be more active in the late stage, which might be associated with the increased demand for nucleotide synthesis and antioxidant defence in the endophytic fungus), and cymbopogone (terpenoids). For the “21d *vs* 28d” comparison (**Figure 6G**), the downregulated metabolites were 16,17-dihydro-16α,17-dihydroxy GA9 (gibberellin), hallactone B (its downregulation again may be related to the metabolic cycle or environmental adaptation strategy of the endophytic fungus; the fungus may reduce the synthesis of this compound in the late stage to conserve resources), and lucidenic acid A (triterpenoids). The upregulated metabolites were 2beta,23-dihydroxy-3beta-[(3-O-alpha-L-rhamnopyranosyl-beta-D-glucopyranuronosyl)oxy]oleana-12-ene-28-oic acid 28-beta-D-glucopyranosyl ester (a complex triterpenoid saponin whose upregulation may be associated with the defensive mechanisms of the endophytic fungus, potentially exhibiting antibacterial and anti-inflammatory bioactivities), 2-propenyl cyclohexanepentanoate (its upregulation may be linked to lipid metabolism in the endophytic fungus, possibly for synthesizing cell membrane components or acting as signalling molecules), and 3S,7,11-trimethyl-6E,10-dodecadien-1-ol (sesquiterpene alcohol).

Through metabolomics analysis of consecutive time points, this study revealed that the metabolic activity of the endophytic fungus NSZJ-CX-22 exhibited stage-specific characteristics, including rapid activation, stable regulation, and stress resuscitation during cultivation. The period 0–7d was designated the metabolic rapid-activation phase, during which the strain efficiently absorbed nutrients to complete cell structure construction and establish the basic metabolic network. The period of 7–21d represented the metabolic stable phase; nutrient depletion and environmental adaptation led to decreased metabolic activity, primary metabolism tended toward a balanced state, and secondary metabolism shifted from stress response to homeostatic regulation. The period of 21–28d was defined as the metabolic stress resuscitation phase; nutrient deprivation triggered the substrate-reutilization mechanism, secondary metabolic pathways were specifically activated, allowing the strain to enhance its environmental adaptability by synthesising bioactive metabolites. Variations in primary metabolites centre on the absorption, storage, and reutilization of nutrients, providing the energy and material basis for the strain’s growth and development. Dynamic changes in secondary metabolites were closely associated with the strain’s environmental adaptation strategies; the stage-specific accumulation of jasmonic acid and its derivatives clearly reflected the fungus’s metabolic transition from stress response to homeostatic regulation.

### 3.5 KEGG pathway enrichment analysis of differential metabolites at different culture time points

To further explore the core metabolic regulatory network of the endophytic fungus NSZJ-CX-22 at different culture time points and clarify the pathway response characteristics mediated by differential metabolites, this study performed KEGG pathway enrichment analysis using all annotated metabolites as the background set. Differential metabolites were mapped to the KEGG pathways. The significance of enrichment was evaluated via the hypergeometric test, and the Benjamini-Hochberg method was applied for P-value correction. Pathways with a corrected P-value (FDR) < 0.05 were defined as significantly enriched. The main enriched core pathways included phenylpropanoid, secondary metabolite, and flavonoid biosynthesis.

These pathways achieve convergence through:(i) Substrate convergence: Acetyl-CoA as common carbon precursor; phenylpropanoid pathway (phenylalanine → cinnamic acid via PAL) provides aromatic skeletons; terpenoid pathway generates FPP; flavonoid pathway utilizes malonyl-CoA—complementary precursor pools for sesquiterpene and 2-(2-phenylethyl)chromone synthesis (Barden et al., 2000; Rasool and Mohamed, 2016; Turjaman et al., 2016; López-Sampson and Page, 2018). (ii) Enzymatic coordination: CHS, TPS, and PKS exhibit temporal synchrony—their substrate-binding domain activities peak concurrently at day 14, achieving directional catalysis and branched metabolism of shared precursors. (iii) Signalling convergence (Barden et al., 2000): jasmonate- and salicylate-related metabolites may represent putative regulatory nodes associated with the timing of secondary metabolism in the fungal substrate culture system; however, the underlying transcriptional regulators remain to be validated by transcriptomic/proteomic evidence. Additionally, amino acid biosynthesis provides nitrogen sources for flavonoid/sesquiterpene synthesis. At the same time, vitamin B6 metabolism serves as a cofactor in tryptophan/tyrosine metabolism, influencing auxin (IAA) and antioxidant production, indirectly supporting secondary metabolism.

**Figure 7:**
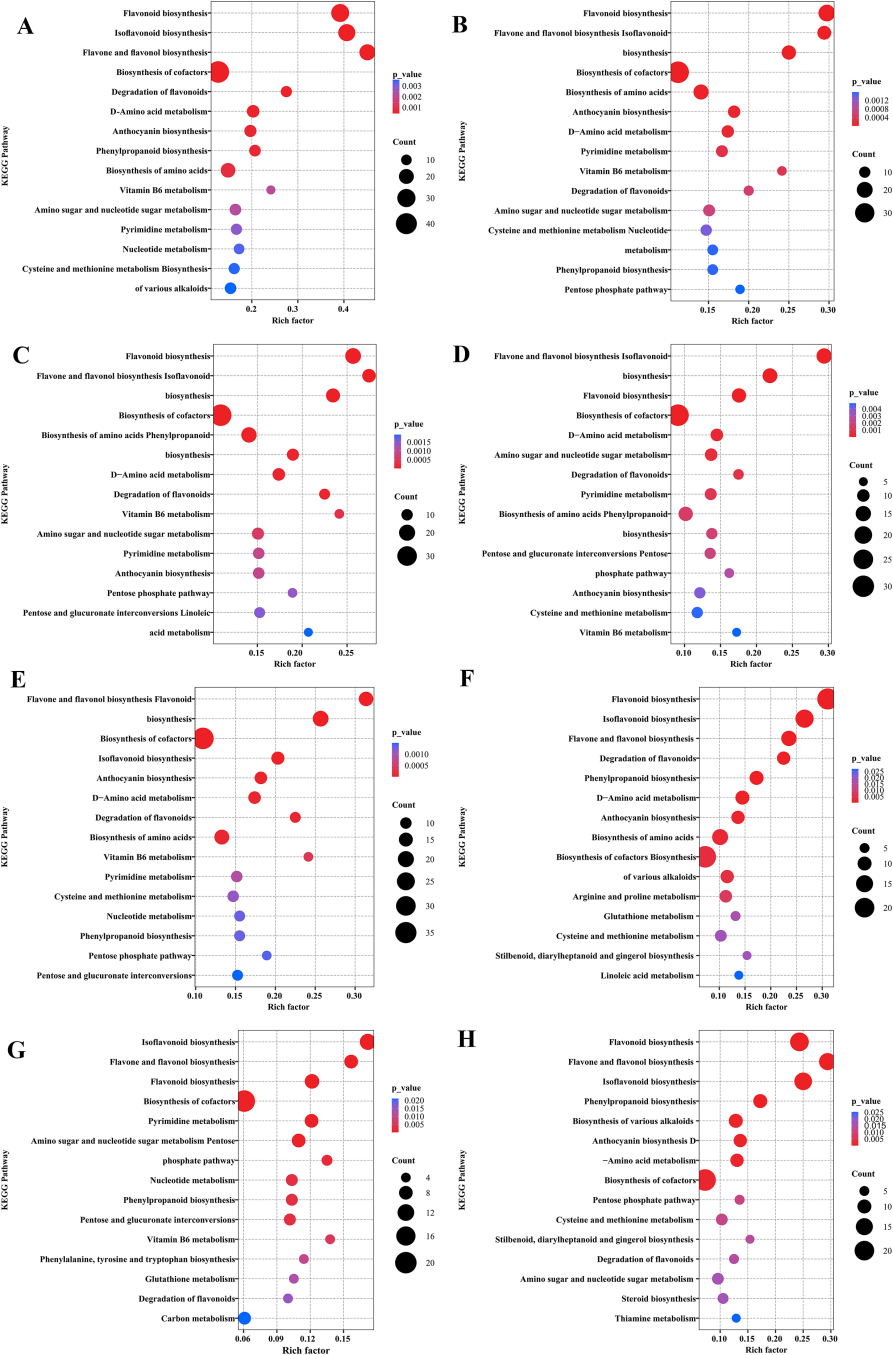
KEGG Enrichment bubble of metabolic pathway analysis. **(A)** KEGG Enrichment bubble of metabolic pathway for the overall comparison group of BM *vs* NSZJ-CX-22. The colour of each bubble indicates the pathway’s significance level (red represents extremely high significance, while blue represents relatively low significance). The size of each bubble denotes the number of differential genes (Count). The horizontal axis represents the enrichment factor, and the vertical axis represents the names of the enriched metabolic pathways. **(B–E)** KEGG Enrichment bubble of metabolic pathway for comparison groups at different culture time gradients, corresponding to BM *vs* 7d (early culture stage, B), BM *vs* 14d (mid-culture stage, **(C)**), BM *vs* 21d (mid-late culture stage, **(D)**), and BM *vs* 28d (late culture stage, **(E)**), respectively. **(F–H)** KEGG Enrichment bubble of metabolic pathway for comparison groups at adjacent culture time points, corresponding to 7d *vs* 14d **(F)**, 14d *vs* 21d **(G)**, and 21d *vs* 28d **(H)**, respectively.

In the overall comparative analysis of “BM *vs* NSZJ-CX-22”, 135 metabolic pathways were enriched. Results from the bubble plot (**Figure 7A**) indicated that flavonoid biosynthesis, isoflavonoid biosynthesis, and flavone and flavonol biosynthesis were the core significantly enriched pathways, characterised by red bubble colour, extremely small P-values, high enrichment factors (Rich factor), and large numbers of differential genes (Count). Biosynthesis of cofactors also exhibited significant enrichment features (with relatively small P-values). Although anthocyanin and phenylpropanoid biosynthesis showed a certain degree of enrichment, with large numbers of differential genes, their significance levels were lower than those of the aforementioned two categories of pathways. In contrast, the biosynthesis of various alkaloids showed low enrichment degree and weak significance (represented by small blue bubbles and low enrichment factors). The above results suggested that the core of metabolic regulation in NSZJ-CX-22 might be concentrated in flavonoid-related secondary metabolic pathways, accompanied by the synergistic activation of cofactor biosynthesis pathways.

To elucidate the regulatory pattern of culture time accumulation across metabolic pathways, four time-gradient paired comparison groups (“BM *vs* 7d, BM *vs* 14d, BM *vs* 21d, BM *vs* 28d”) were established, yielding 130, 128, 124, and 129 enriched metabolic pathways, respectively. The slight fluctuation in the number of pathways throughout the entire culture period suggested that the strain’s metabolic activity remained persistent and stable. Combined with the top 10 differential metabolites ranked by VIP values identified via multivariate statistical analysis, the association between pathways and differential metabolites at each stage, as well as the core metabolic regulatory characteristics, were further clarified as follows: Early cultivation stage (BM *vs* 7d, **Figure 7B**), flavonoid-related pathways (flavonoid biosynthesis, flavone and flavonol biosynthesis, isoflavonoid biosynthesis) were the core significantly enriched pathways. In combination with the characteristics of differential metabolites described in Section 3.3, the upregulation of (+)-costunolide and delta-tetradecalactone indicated the enhanced activity of terpenoid biosynthesis pathways and PKS biosynthesis pathways, which was consistent with the activation feature of flavonoid-related secondary metabolic pathways revealed by pathway enrichment analysis. In addition, nucleotide metabolism and phenylpropanoid biosynthesis showed the weakest enrichment in this group, which also echoed the downregulation trend of 3,4-dimethoxycinnamyl alcohol, a lignin precursor compound among the differential metabolites. Mid-cultivation stage (BM *vs* 14d, BM *vs* 21d, **Figures 7C, D**). For the “BM *vs* 14d” group, flavonoid-related pathways and phenylpropanoid biosynthesis remained significantly enriched (with phenylpropanoid biosynthesis showing a moderate enrichment level). The top 3 upregulated VIP metabolites in this stage were methylnissolin-3-O-glucoside, tremetone, and (15Z)-9,12,13-trihydroxy-15-octadecenoic acid, which matched the activation characteristics of secondary metabolic pathways such as flavonoid and phenylpropanoid biosynthesis. This reflected that after entering the mid-cultivation stage, the strain further enhanced secondary metabolic activity to adapt to environmental changes. For the “BM *vs* 21d” group, flavone and flavonol biosynthesis was the most significantly enriched pathway (with the highest enrichment factor and the smallest P-value), while pathways such as cofactor biosynthesis showed a moderate degree of enrichment. The significant upregulation of secondary metabolites, including the alkaloid atherosperminine and the terpenoid neotriptophenolide in this stage, was consistent with the sustained enrichment of flavonoid-related pathways and the gradual activation of alkaloid biosynthesis pathways. These results demonstrated that the defensive function of secondary metabolism was further strengthened during the mid-to-late cultivation period, and the regulation of metabolic pathways became more specific, with the core defensive secondary metabolic functions being further enhanced. In addition, the cysteine and methionine metabolism and vitamin B6 metabolism pathways showed low enrichment levels, consistent with the regulatory variation characteristics of amino acid-derived substances. At the late cultivation stage (BM *vs* 28d, **Figure 7E**), flavonoid-related pathways, cofactor biosynthesis, and amino acid biosynthesis showed high enrichment levels, whereas the pentose phosphate pathway and pentose and glucuronate interconversions showed the lowest enrichment. This observation highlighted the regulatory characteristics of carbohydrate-derived metabolites among the differential metabolites at this stage, indicating that the strain’s metabolic regulation in the late cultivation period focused on the biosynthesis of core secondary metabolites and on optimizing energy metabolism.

To further trace the dynamic transition pattern of metabolic pathways between adjacent culture time points, four paired comparison groups of adjacent time points (“BM *vs* 7d, 7d *vs* 14d, 14d *vs* 21d, 21d *vs* 28d”) were established, yielding 130, 125, 97, and 113 enriched metabolic pathways, respectively. The overall downward trend in the number of pathways indicated that, as cultivation progressed, the metabolic regulation of NSZJ-CX-22 gradually shifted its focus to core pathways. Combined with the top 10 differential metabolites ranked by VIP values identified via multivariate statistical analysis, the enrichment characteristics of metabolic pathways in adjacent stages were as follows. For the “7d *vs* 14d” group (**Figure 7F**), the core pathways significantly enriched included flavonoid biosynthesis, isoflavonoid biosynthesis, and flavone and flavonol biosynthesis. Flavonoid degradation, phenylpropanoid biosynthesis, and D-amino acid metabolism also exhibited significant enrichment. In combination with the variations in differential metabolites during this stage, such as the decrease in the alkaloid ergometrine and the increase in the glycoside 2-phenylethyl 2-O-[(2S,3R,4R)-3,4-dihydroxy-4-(hydroxymethyl)tetrahydro-2-furanyl]-beta-D-glucopyranoside, these results reflected that the strain gradually shifted from basic defence and energy storage in the early cultivation stage to secondary metabolism enhancement and efficient energy utilization in the mid-stage, which was consistent with the dynamic changes in pathway enrichment levels. For the “14d *vs* 21d” group (**Figure 7G**), isoflavonoid biosynthesis showed the highest enrichment degree and the strongest significance. Flavone and flavonol biosynthesis, flavonoid biosynthesis, and cofactor biosynthesis were all significantly enriched. The correlation characteristics between differential metabolites and pathways indicated that when the strain entered the mid-to-late cultivation stage, the core of metabolic regulation shifted from oxidative stress to the synthesis of specific secondary metabolites and the enhancement of core metabolic pathways. This was exemplified by the upregulation of Hallactone B (associated with the terpenoid synthesis pathway) and 6-Phosphogluconic acid (an intermediate product of the pentose phosphate pathway). For the “21d *vs* 28d” group (**Figure 7H**), flavonoid-related pathways, phenylpropanoid biosynthesis, and biosynthesis of various alkaloids were all highly enriched. The differential metabolites in this stage were dominated by upregulation of specific secondary metabolites, suggesting that the strain’s metabolic regulation at the late cultivation stage focused on the synthesis of core defensive secondary metabolites. This was consistent with fewer enriched pathways and sustained activation of core pathways.

**Figure 8:**
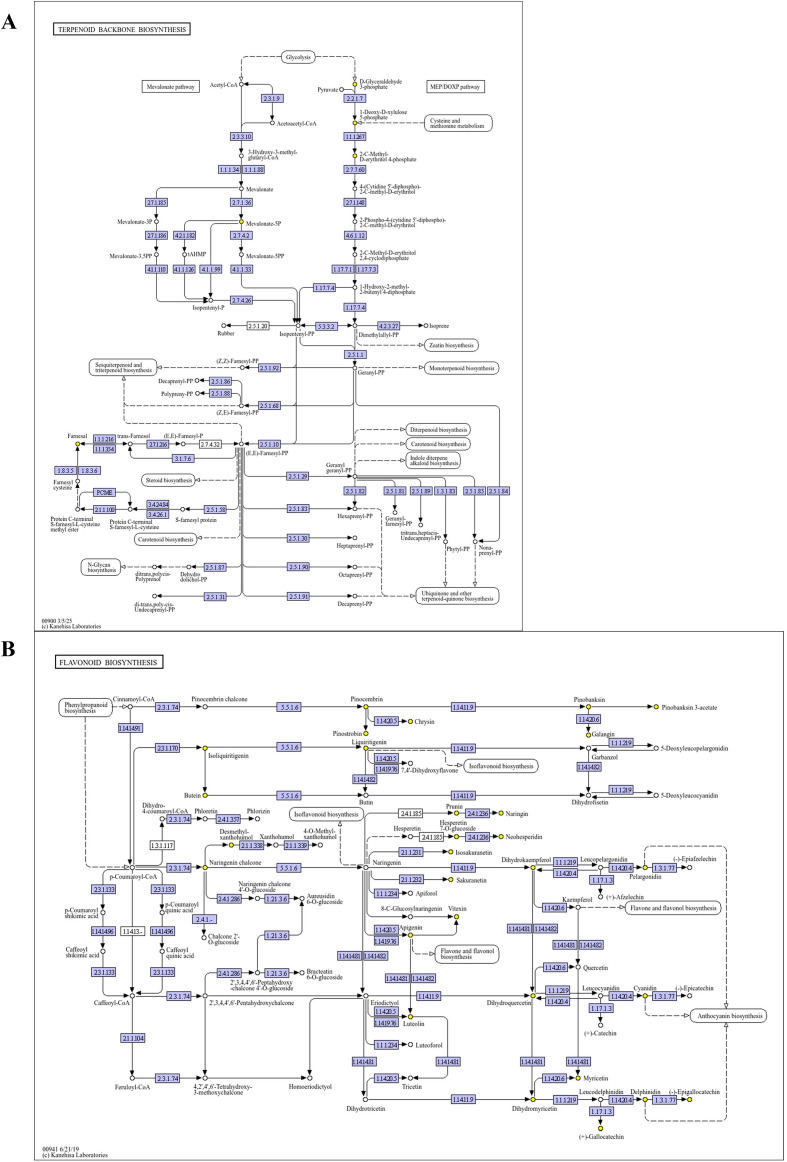
Core secondary metabolic pathways of Aquilaria sinensis in the pure culture model. Terpenoid backbone biosynthesis pathway **(A)** and flavonoid biosynthesis pathway **(B)**. Red indicates key pathway names (terpenoid backbone biosynthesis, MVA pathway, MEP/DOXP pathway, flavonoid biosynthesis, phenylpropanoid pathway); blue indicates core precursor substances (acetyl-CoA, pyruvate, glyceraldehyde-3-phosphate, IPP, DMAPP, FPP, GPP, p-coumaroyl-CoA); green indicates key intermediates and end products (chalcones, flavanones, flavonols, proanthocyanidins, sesquiterpenes, triterpenes, diterpenes); arrows indicate the direction of metabolic reactions.

In a pure culture model using *Aquilaria sinensis* wood chips as the substrate, the coordinated activation and substrate-allocation characteristics of flavonoid and terpenoid metabolic pathways together constitute the core regulatory pattern of secondary metabolism in A. sinensis. This pattern can be systematically elucidated through KEGG pathway enrichment and bubble-plot analyses. As the key upstream pathway for terpenoid production in A. sinensis, terpenoid backbone biosynthesis (**Figure 8A**) is jointly driven by the mevalonate (MVA) pathway and the methylerythritol phosphate (MEP/DOXP) pathway. These pathways start from acetyl−CoA and from pyruvate/glyceraldehyde−3−phosphate, respectively, and ultimately generate two universal C5 precursors— isopentenyl pyrophosphate (IPP) and dimethylallyl pyrophosphate (DMAPP). These are further condensed into longer-chain precursors such as farnesyl pyrophosphate (FPP) and geranyl pyrophosphate (GPP), which provide the carbon skeletons for the biosynthesis of defence-related terpenoids, including sesquiterpenes, triterpenes, and diterpenes. Meanwhile, flavonoid biosynthesis (**Figure 8B**) uses p−coumaroyl−CoA, a key precursor derived from the phenylpropanoid pathway, and proceeds through intermediate nodes such as chalcones and flavanones, ultimately diverging into functional subtypes including flavonols, flavanones, and proanthocyanidins, forming another major branch that supports antioxidant capacity and chemical defence in A. sinensis.

Under this abiotic-stress model using the A. sinensis wood-chip medium, these two pathways exhibit a pattern of synergistic activation driven by substrate competition. On the one hand, terpenoid and flavonoid pathways share upstream carbon sources from primary metabolism (e.g., acetyl−CoA, malonyl−CoA, and phenylalanine), as shown by the bubble showing certain antagonistic tendencies, reflecting trade-offs in allocating limited carbon resources across different secondary-metabolic branches. On the other hand, as core components of secondary defence metabolism in A. sinensis, both pathways are globally upregulated upon induction by the wood-chip substrate, as evidenced by significant enrichment of “terpenoid backbone biosynthesis” and “flavonoid biosynthesis” in KEGG enrichment analysis. Moreover, the expression of key enzyme genes (e.g., HMGR and DXR in the terpenoid pathway; CHS and FNS in the flavonoid pathway) is positively correlated with metabolite abundance, together forming a secondary-metabolic defence network that enables A. sinensis to cope with substrate-induced stress. This “global synergy with local competition” regulatory mode not only ensures rapid accumulation of diverse defence-related secondary metabolites in the wood-chip substrate, but also achieves efficient utilization of metabolic resources via substrate allocation, thereby providing a key regulatory basis for the biosynthesis of characteristic secondary metabolites in A. sinensis (e.g., sesquiterpenes and flavonols).

In summary, pathway enrichment of the endophytic fungus NSZJ−CX−22 is centred on flavonoid-related pathways (flavonoid biosynthesis, flavone and flavonol biosynthesis, and isoflavonoid biosynthesis), with cofactor biosynthesis and amino-acid biosynthesis serving as important associated pathways. The significant enrichment of these core pathways persists throughout the entire cultivation process and shows dynamic changes across adjacent time points. Further association analysis between differential metabolites and pathways reveals stage-specific metabolic regulatory features: during the early stage of cultivation (0–7 d), activation of terpenoid- and polyketide-related secondary metabolism and the establishment of basic energy reserves are predominant; during the mid stages (7–14 d and 14–21 d), metabolism is characterized by strengthened secondary metabolism, oxidative-stress responses, and optimized energy utilization; and during the late stage (21–28 d), metabolism shifts toward the biosynthesis of core defence-related secondary metabolites (e.g., triterpenoid saponins and alkaloids). Metabolic activity of the strain remains sustained and stable throughout the cultivation period, whereas the overall number of enriched metabolic pathways declines as cultivation progresses, indicating that metabolic regulation gradually converges on core pathways. Clear stage-specific differences are observed in pathway–metabolite associations across cultivation phases. These results provide key evidence to clarify the core metabolic activities of NSZJ−CX−22 at different cultivation stages, the functional orientation of differential metabolites, and the underlying mechanisms of metabolic regulation.

### 3.6 Composition and dynamic variation of volatile metabolites at different culture time points

**Figure 9:**
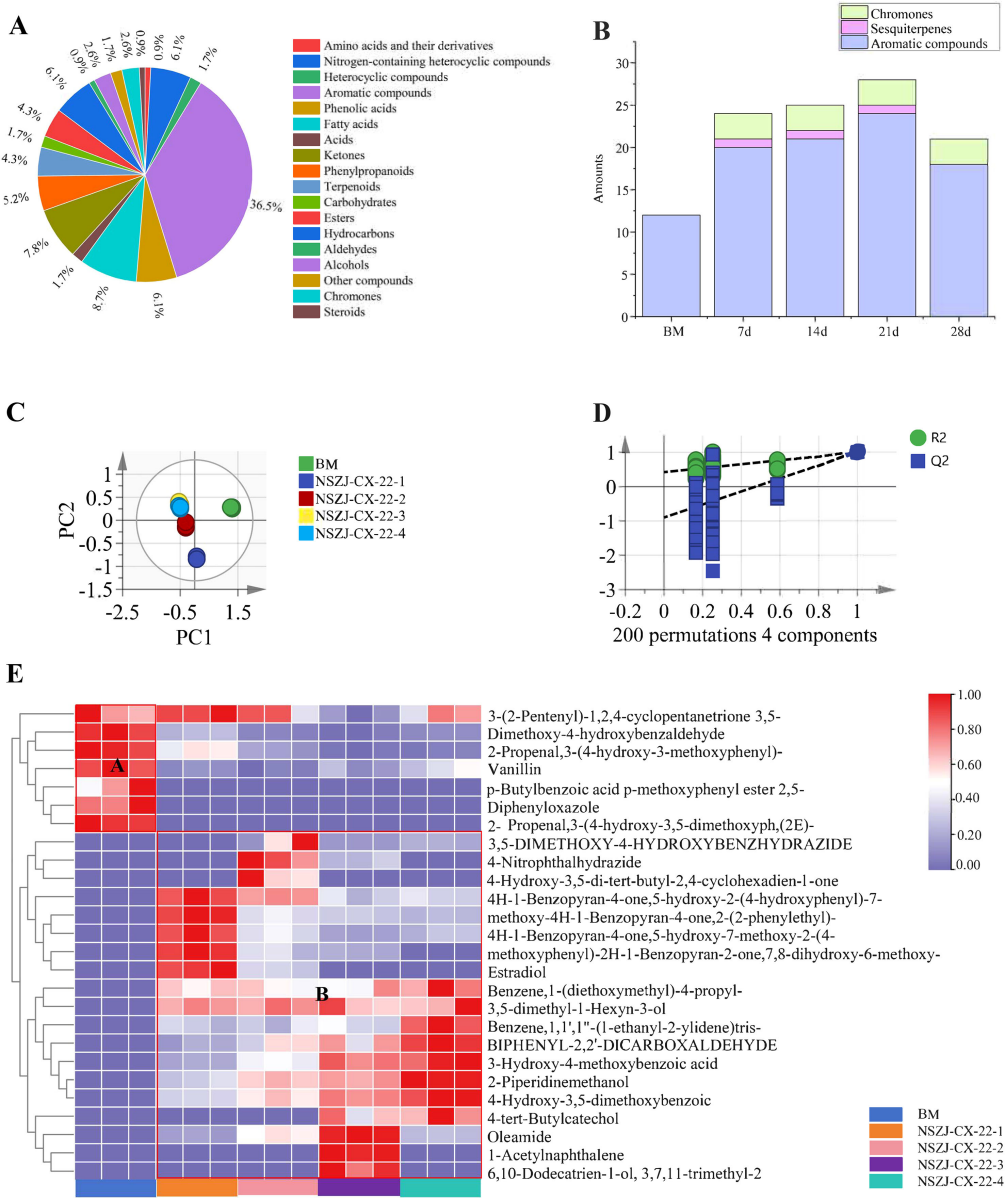
Identification and multivariate statistical analysis of volatile metabolites of strain NSZJ-CX-22 at different culture time points. **(A)** Chemical classification distribution of volatile metabolites. **(B)** Dynamic trends in the variation of agarwood characteristic aroma-related compounds (sesquiterpenoids, chromones, and aromatic compounds) over culture time. **(C)** Principal component analysis (PCA) score plot of volatile metabolites in samples at different culture time points. **(D)** 200-time permutation test results of the orthogonal partial least squares-discriminant analysis (OPLS-DA) model. **(E)** Time-series heatmap and cluster analysis results of key differential metabolites, clearly showing the metabolic characteristics of different regions: Region A represents the characteristic region of the blank medium, while Region B represents the characteristic region of fungal metabolism, exhibiting significant stage-specific variations.

To elucidate the dynamic variation in volatile metabolites produced by strain NSZJ-CX-22 across different cultivation stages, GC–MS was employed for efficient separation and identification. Mass spectral matching was performed using Agilent 5975C MSD MassHunter software, and compound structural matching was performed against the NIST11 database. The relative content of each compound was calculated using the area-normalisation method. A total of 116 volatile metabolites were identified, belonging to 17 chemical categories (**Figure 9A**). Among these, aromatic compounds (42 species), terpenoids (5 species), and chromones (3 species) were classified as key aroma components. The major categories also included fatty acids (10 species), ketones (9 species), phenolic acids (7 species), and phenylpropanoids (6 species). Minor categories involved flavonoids (1 species) and steroids (1 species), among others. These findings indicate that the strain NSZJ-CX-22 is capable of synthesising a diverse array of small volatile molecules in the *A. sinensis* medium, with a significant enrichment of aromatic compounds and terpenoids, thereby providing a chemical basis for its induction of agarwood aroma formation. Notably, sesquiterpenoids, chromone derivatives, and aromatic compounds were synthesised in a stage-specific manner following strain inoculation. This discovery not only explains why the agarwood aroma induced by endophytic fungi is more distinctive and intense than that induced by chemical inducers, but also corroborates the strain’s application potential as a high-quality biological inducer. The distinctive aroma of agarwood is typically determined by its chemical composition, including the quantity and composition of sesquiterpenoids, chromones, and aromatic compounds, which exhibit dynamic changes with the progression of culture time (**Figure 9B**).

Results of multivariate statistical analysis indicated that the volatile metabolic profile of strain NSZJ-CX-22 exhibited a significant time-dependent pattern. Results of PCA (**Figure 9C**) showed a clear separation trend among samples at different culture time points. Notably, samples collected on the 21d and 28d clustered most closely, suggesting a high similarity in their metabolic profiles. In contrast, these samples exhibited significant differences from those collected on the 7d, indicating that the metabolic reprogramming process in the late cultivation stage dominated the dynamic changes in the metabolic profile, leading to metabolic characteristics deviating from the early-stage state. Based on the VIP values derived from the OPLS-DA model and the P-values from univariate statistical analysis, crucial differential metabolites were successfully screened and identified. Results of 200 permutation tests (**Figure 9D**) showed that the Q2 intercept of the model was negative, indicating that the model had stable fitting performance and good predictive ability, and thus could be effectively applied to the analysis and discrimination of metabolite differences across different culture stages.

A total of 26 key differential metabolites were identified, covering multiple chemical categories including aromatic compounds (5 species), phenylpropanoids (3 species), and flavonoids (3 species). Cluster analysis of the time-series heatmap (**Figure 9E**) clearly visualised the metabolic characteristics of different regions. Region A (blank medium characteristic region) was enriched with substrates such as phenylpropanoids (e.g., 3,5-dimethoxy-4-hydroxybenzaldehyde) and nitrogen-containing heterocyclic compounds. These substrates exhibited a continuous consumption trend over prolonged cultivation, indicating that they participated in the growth and metabolic processes of the strain and served as carbon and nitrogen sources, or metabolic precursors. Region B (fungal metabolism characteristic region) exhibited distinct stage-specific variations. Early stage (0–7d): Chromone derivatives (e.g., 2-(2-phenylethyl)chromone) accumulated rapidly. This is presumed to be a protective mechanism in which chromone derivatives act as antioxidants to mitigate oxidative stress, ensuring the initial growth of the strain. Notably, environmental changes and endogenous metabolic activities during the early growth phase are likely to generate reactive oxygen species, and the rapid accumulation of chromone derivatives provides an effective defence against such oxidative damage. Mid stage (14–21d): The contents of sesquiterpene precursors (e.g., 6,10-dodecadien-1-ol) and aromatic compounds (e.g., 1-acetylnaphthalene) were significantly upregulated, indicating the activation of the sesquiterpene biosynthetic pathway. Sesquiterpenoids typically play versatile roles in fungal physiological processes, including acting as signalling molecules to regulate fungal growth, development, and interactions with the external environment. Meanwhile, variation in aromatic compound content may be associated with the sesquiterpene biosynthetic pathway or participate in other secondary metabolic processes. Late stage (28d): phenolic acids (e.g., 3-hydroxy-4-methoxybenzoic acid) and nitrogen-containing compounds (e.g., 2-piperidinemethanol) increased significantly, suggesting the enhanced catalytic activity of CYP450-mediated modification reactions. CYP450 is a class of important oxidoreductases that can catalyse various chemical reactions for metabolite modification. The accumulation of phenolic acids and nitrogen-containing compounds in the late stage is highly likely to be involved in synergistic defensive metabolic activities. Phenolic acids possess certain antibacterial activities, while nitrogen-containing compounds may also exert effects in antibacterial defence. Their coordinated accumulation probably represents an active defensive strategy adopted by the strain in the late growth phase to cope with potential external threats, such as pathogen infection or competition from other microorganisms.

In summary, the volatile metabolism of the endophytic fungus NSZJ-CX-22 in the *A. sinensis* medium exhibited distinct three-stage characteristics. Nutrient transformation phase (0–7d): The strain consumed substrates to synthesise early metabolites such as flavonoids. Aroma biosynthesis phase (14–21d): Sesquiterpene precursors and aromatic compounds accumulated explosively, laying the chemical foundation for the characteristic agarwood aroma. Defence enhancement phase (21–28d): Phenolic acids and nitrogen-containing compounds were generated via CYP450-mediated oxidative modification, indicating enhanced late-stage oxidative modification and nitrogen-containing metabolite accumulation, which likely contribute to fungal stress tolerance and diversification of aroma-relevant volatiles under substrate nutrient limitation. This model not only clarifies the chemical components responsible for the unique agarwood aroma induced by the fungus but also highlights a temporally programmed metabolic redistribution strategy in the fungus–substrate culture system. Specifically, NSZJ-CX-22 appears to coordinate early antioxidant-like chromone accumulation, mid-stage terpene/aromatic volatile enrichment, and late-stage P450-associated derivatization, thereby jointly shaping aroma complexity and sustaining fungal adaptation during prolonged cultivation.

## 4 Conclusion

This study systematically resolved the time-dependent metabolic programming of the endophytic fungus *Arthrinium* sp. NSZJ-CX-22 in a non-living *Aquilaria sinensis* sawdust substrate model (where sawdust served only as a cultivation matrix rather than living host tissue), establishing a “precursor pool–pathway convergence–temporal programming” framework for fungus-driven production of agarwood-characteristic metabolites. By integrating LC-MS/MS and GC-MS, we annotated 14,784 metabolites, including 118 agarwood-characteristic sesquiterpene skeletons and 2-(2-phenylethyl)chromones, and showed that flavonoid/phenylpropanoid/terpenoid-related pathways converge through shared precursors (acetyl-CoA and malonyl-CoA) and coordinated branch-point enzymes (CHS, TPS, PKS), with the strongest pathway activation occurring around day 14. Importantly, quantitative correlations linked precursor pool contraction to product accumulation (|r| ≈ 0.63–0.74, FDR<0.05), supporting a timed transition from primary substrate utilization to secondary metabolite biosynthesis, followed by late-stage P450-associated derivatization that increases chemical diversity; together, these results provide biological meaning to the observed metabolic shift as an intrinsic fungal strategy to reallocate carbon/nitrogen resources and maintain secondary metabolism under nutrient limitation, rather than a host-interaction response. From an application perspective, the identified temporal windows translate directly into process guidance for industrial production of aroma-relevant metabolites: (i) prioritize harvesting of sesquiterpene-rich fractions during 7–14 d (when terpene biosynthesis is most active), (ii) avoid the metabolic trough around ~21 d for chromone-oriented production and instead harvest before 14 d or after 28 d depending on target profiles, and (iii) leverage the 21–28 d phase to enhance oxidative tailoring and aroma layering by controlling oxygen supply and pH stability, potentially combined with segmented cultivation or fed-batch feeding to sustain precursor availability and improve yield consistency. Relative to prior agarwood biosynthesis studies that often emphasize pathway identification at single time points or infer mechanisms from plant–microbe interaction contexts, our work advances current knowledge by (a) providing a time-resolved, system-level map of fungal secondary metabolism in a defined substrate culture, (b) quantitatively linking precursor depletion to agarwood-characteristic metabolite accumulation, and (c) integrating non-volatile metabolomics with volatile profiling to connect metabolic reprogramming to aroma outcomes, thereby offering a more engineerable basis for standardized, scalable, and potentially more sustainable production strategies.

## Data Availability

The raw data supporting the conclusions of this article will be made available by the authors, without undue reservation.
