# Supplementary material for: Dynamic regulation of secondary metabolites and agarwood aroma compounds in Aquilaria sinensis by the endophytic fungus NSZJ-CX-22 revealed through metabolomics and GC–MS
Source: Front Plant Sci. 2026 Apr 10;17:1750077. doi: 10.3389/fpls.2026.1750077 (PMC13106441; doi:10.3389/fpls.2026.1750077)
Supplement: Supplementary file 1 [file DataSheet1.docx]

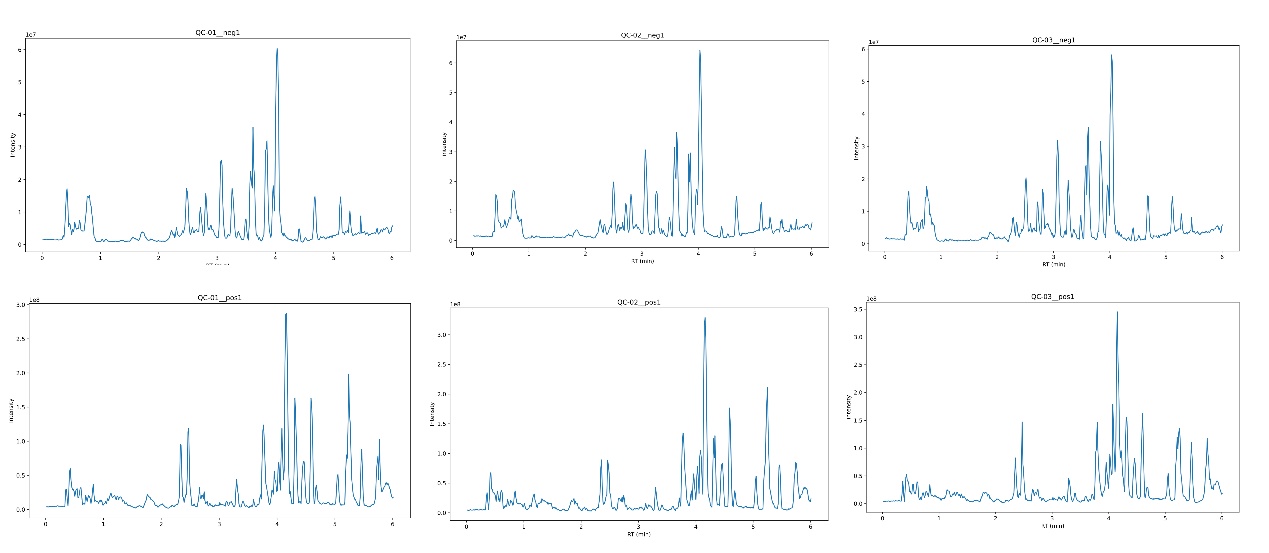


Fig. A. Quality control assessment of sample detection.Total ion chromatograms (TICs) of mixed samples and the overlaid TICs of QC samples are shown to evaluate analytical stability and consistency across measurements.


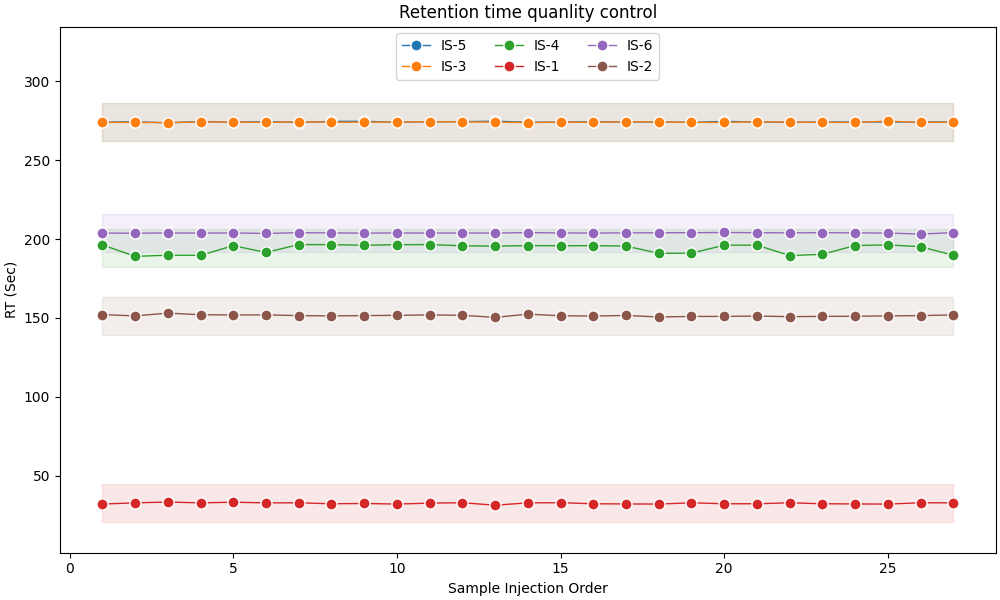


Fig. B. Retention time QC plot for the internal standard.The retention times of the internal standard in all samples and QC samples deviated by less than ±12 s, demonstrating stable instrument performance throughout the analytical process.





Fig. C. QC sample correlation analysis.Correlation analysis of QC samples showed coefficients close to 1, demonstrating high analytical stability and confirming the reliability of the metabolomic data.


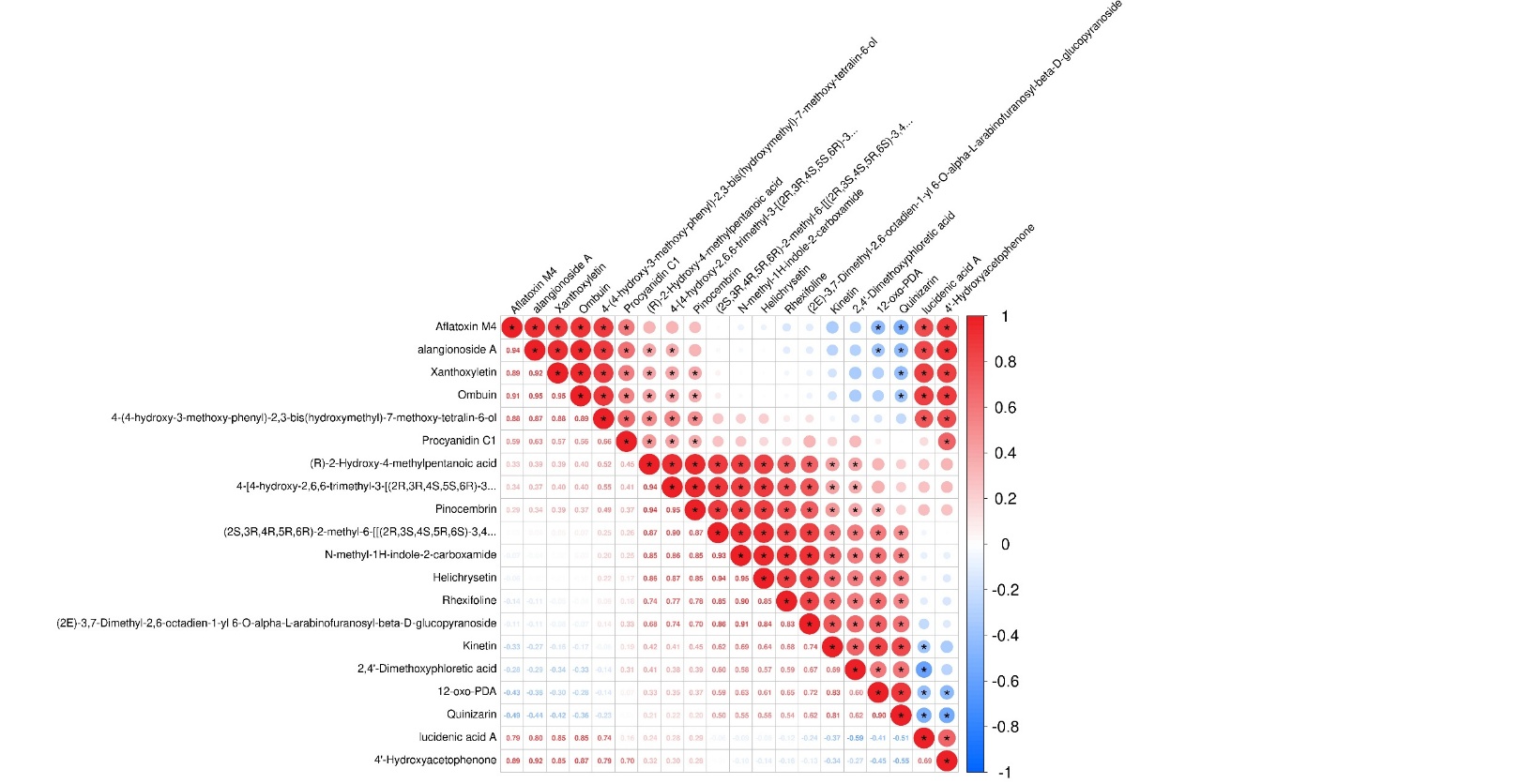


Fig. D. Pearson correlation analysis within sample groups.Pearson correlation coefficients for samples within each group were close to 1, indicating strong biological reproducibility and supporting the reliability of the identified differential metabolites.


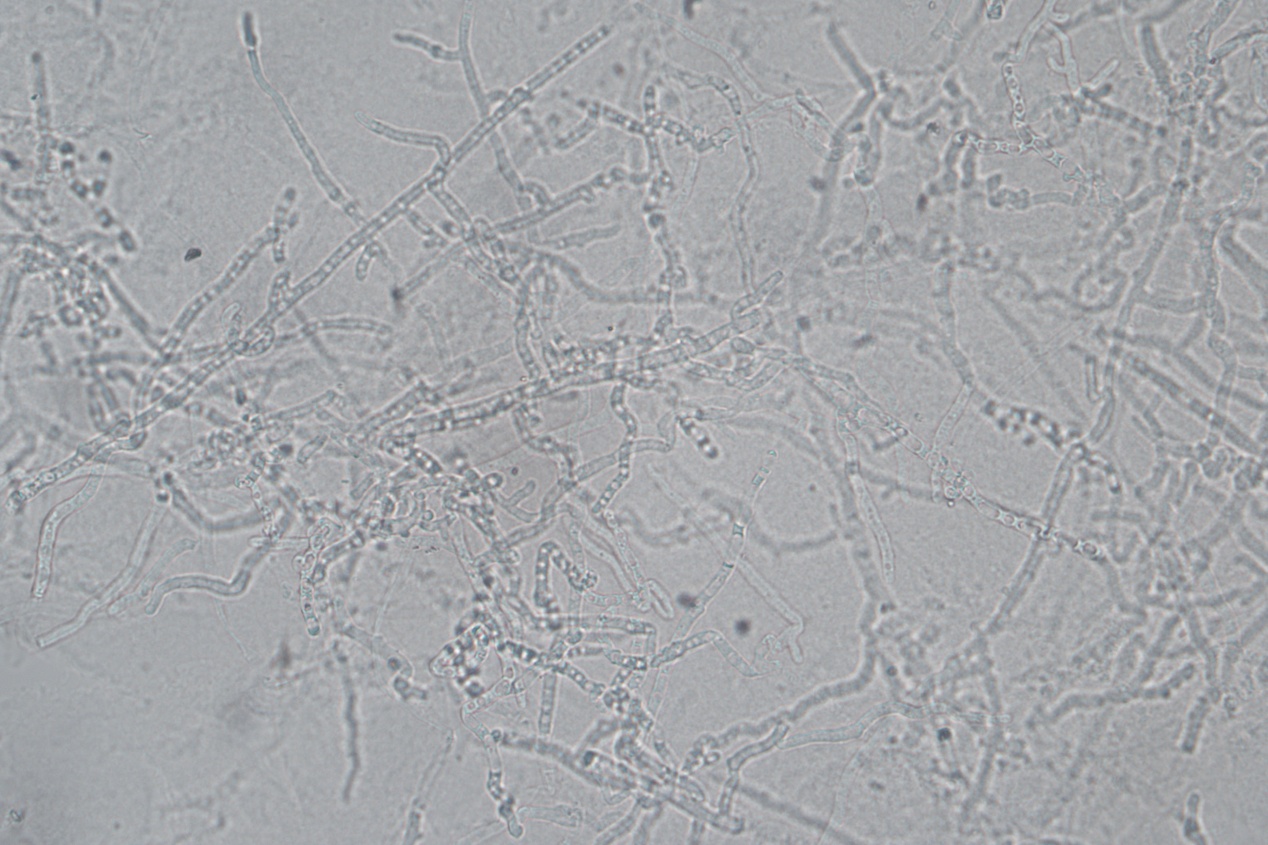


Fig.E. Micrograph of endophytic fungus NSZJ-CX-22.
